# Extensive Thioautotrophic Gill Endosymbiont Diversity within a Single *Ctena orbiculata* (Bivalvia: Lucinidae) Population and Implications for Defining Host-Symbiont Specificity and Species Recognition

**DOI:** 10.1128/mSystems.00280-19

**Published:** 2019-08-27

**Authors:** Shen Jean Lim, Louie Alexander, Annette Summers Engel, Audrey T. Paterson, Laurie C. Anderson, Barbara J. Campbell

**Affiliations:** a Department of Biological Sciences, Clemson University, Clemson, South Carolina, USA; b Department of Earth and Planetary Sciences, University of Tennessee—Knoxville, Knoxville, Tennessee, USA; c Department of Geology and Geological Engineering, South Dakota School of Mines & Technology, Rapid City, South Dakota, USA; University of California, Riverside

**Keywords:** host-microbe interactions, lucinid, metagenomics, metatranscriptomics, symbiosis

## Abstract

Seagrass-dwelling members of the bivalve family Lucinidae harbor environmentally acquired gill endosymbionts. According to previous studies, lucinid symbionts potentially represent multiple strains from a single thioautotrophic gammaproteobacterium species. This study utilized genomic- and transcriptomic-level data to resolve symbiont taxonomic, genetic, and functional diversity from Ctena orbiculata endosymbiont populations inhabiting carbonate-rich sediment at Sugarloaf Key, FL (USA). The sediment had mixed seagrass and calcareous green alga coverage and also was colonized by at least five other lucinid species. Four coexisting, thioautotrophic endosymbiont operational taxonomic units (OTUs), likely representing four strains from two different bacterial species, were identified from *C. orbiculata*. Three of these OTUs also occurred at high relative abundances in the other sympatric lucinid species. Interspecies genetic differences averaged about 5% lower at both pairwise average nucleotide identity and amino acid identity than interstrain differences. Despite these genetic differences, *C. orbiculata* endosymbionts shared a high number of metabolic functions, including highly expressed thioautotrophy-related genes and a moderately to weakly expressed conserved one-carbon (C_1_) oxidation gene cluster previously undescribed in lucinid symbionts. Few symbiont- and host-related genes, including those encoding symbiotic sulfurtransferase, host respiratory functions, and host sulfide oxidation functions, were differentially expressed between seagrass- and alga-covered sediment locations. In contrast to previous studies, the identification of multiple endosymbiont taxa within and across *C. orbiculata* individuals, which were also shared with other sympatric lucinid species, suggests that neither host nor endosymbiont displays strict taxonomic specificity. This necessitates further investigations into the nature and extent of specificity of lucinid hosts and their symbionts.

**IMPORTANCE** Symbiont diversity and host/symbiont functions have been comprehensively profiled for only a few lucinid species. In this work, unprecedented thioautotrophic gill endosymbiont taxonomic diversity was characterized within a *Ctena orbiculata* population associated with both seagrass- and alga-covered sediments. Endosymbiont metabolisms included known chemosynthetic functions and an additional conserved, previously uncharacterized C_1_ oxidation pathway. Lucinid-symbiont associations were not species specific because this *C. orbiculata* population hosted multiple endosymbiont strains and species, and other sympatric lucinid species shared overlapping symbiont 16S rRNA gene diversity profiles with *C. orbiculata*. Our results suggest that lucinid-symbiont association patterns within some host species could be more taxonomically diverse than previously thought. As such, this study highlights the importance of holistic analyses, at the population, community, and even ecosystem levels, in understanding host-microbe association patterns.

## Abstract (precis)

Symbiont diversity and host/symbiont functions have been comprehensively profiled for only a few lucinid species. In this work, unprecedented thioautotrophic gill endosymbiont taxonomic diversity was characterized within a *Ctena orbiculata* population associated with both seagrass- and alga-covered sediments. Endosymbiont metabolisms included known chemosynthetic functions and an additional conserved, previously uncharacterized C_1_ oxidation pathway. Lucinid-symbiont associations were not species specific because this *C. orbiculata* population hosted multiple endosymbiont strains and species, and other sympatric lucinid species shared overlapping symbiont 16S rRNA gene diversity profiles with *C. orbiculata*. Our results suggest that lucinid-symbiont association patterns within some host species could be more taxonomically diverse than previously thought. As such, this study highlights the importance of holistic analyses, at the population, community, and even ecosystem levels, in understanding host-microbe association patterns.

## INTRODUCTION

Chemolithoautotrophic symbiosis, where symbiotic bacteria use inorganic chemical energy for the synthesis of organic compounds that benefit their hosts, is prevalent in marine bivalves, including the Lucinidae (1, 2). All extant lucinid species examined to date host chemosynthetic bacterial endosymbionts belonging to the class *Gammaproteobacteria* within specialized epithelial gill cells known as bacteriocytes (3). Lucinid gill endosymbionts possess a diverse and varied suite of functions, including thioautotrophy (4), aerobic respiration (5), assimilatory and dissimilatory nitrate reduction (6), mixotrophy (7, 8), hydrogenotrophy (7, 8), and diazotrophy (7, 9), which enable lucinids to colonize otherwise uninhabitable conditions (10). Specifically, lucinids burrow into sediments where oxygen is available from the oxic water column and dissolved sulfide is present due to the decomposition of organic matter via sulfate reduction (2, 3, 11). In modern seagrass habitats, a three-way symbiotic association among bacteria, lucinids, and seagrass has been proposed (11, 12). Lucinid hosts acquire oxygen for respiration from oxygenated water surrounding seagrass roots, and the thioautotrophic endosymbionts oxidize sulfide while fixing inorganic carbon for the host (12). Seagrass growth is promoted because the endosymbionts remove sulfide and have the potential to fix nitrogen (7, 11, 12). This symbiotic system likely developed in the late Cretaceous period, based on the correspondence of adaptive radiation of shallow marine lucinid species in the fossil record to the evolutionary first appearances of marine angiosperms, including seagrasses (13).

Lucinid gill endosymbionts are related to a larger group of diverse marine thioautotrophic symbionts (1). However, unlike chemosymbiotic Solemyidae and Vesicomyidae bivalves, where vertical or mixed symbiont transmission has been observed (14–16), lucinids studied to date acquire their endosymbionts environmentally (17–19). Based on their 16S rRNA gene sequences, lucinid endosymbionts occur in three distinct clades, with the largest (clade A) originating from hosts that primarily inhabit sediments from seagrass beds and two (clades B and C) originating from lucinids that are more likely to inhabit sediments from mangrove-rich areas (20). It is possible that each clade represents a separate species (8), although an insufficient number of lucinid species has been examined at a sufficiently high genetic resolution to test this hypothesis. At present, low to no variability among clade A lucinid endosymbiont 16S rRNA gene sequences suggests that this group belongs to a single species (20–23). Earlier studies of gill thioautotrophic endosymbionts from lesser Antillean lucinid hosts, including Ctena imbricatula (referred to as Codakia orbiculata in reference 21), Codakia orbicularis, Parvilucina pectinella (referred to as Codakia pectinella in reference 21), Anodontia alba, Divalinga quadrisulcata (referred to as Divaricella quadrisulcata in reference 21), and Lucina roquesana (referred to as Linga pensylvanica in reference 21), show identical 16S rRNA gene sequences (22, 23) and the capability of infecting aposymbiotic *Codakia orbicularis* juveniles (24). Recent reanalysis of these thioautotrophic endosymbionts using five other marker genes (instead of the slow-evolving 16S rRNA gene) revealed that intraspecific symbiont strain diversity in the population was shaped by host geographic location (25). Strain-specific symbiont acquisition was also observed in the same study (25), where starved *Ctena imbricatula* individuals (referred to as *Codakia orbiculata* in reference 21) could reacquire only the exact symbiont strain that they initially hosted before starvation. This suggests that, unlike undifferentiated naive bacteriocytes, mature bacteriocytes can possibly recognize and select specific chemolithoautotrophic symbiont strains (24, 25).

In spite of these findings, species- and strain-level diversity within lucinid endosymbiont clades remains unverified for most lucinid species. To date, endosymbiont functions for two clade A endosymbionts, “*Candidatus* Thiodiazotropha endolucinida” from *Codakia orbicularis* (9) and “*Ca.* Thiodiazotropha endoloripes” from Loripes orbiculatus (syn = Loripes lucinalis) (7), as well as for the clade C endosymbiont from mangrove-dwelling Phacoides pectinatus (8), have been investigated using omics approaches. However, limited attention was given to species- and strain-level diversity among the endosymbiont populations. Information about endosymbiont species- and strain-level diversity will be useful for interhost and interpopulation comparisons and could provide new insight into host-symbiont association patterns and possibly biogeographical or environmental drivers of diversity among free-living bacteria in the environment, prior to being acquired by the host or becoming part of the gill microbiome.

In this study, we focused on characterizing the taxonomic, genetic, and functional composition of endosymbiont communities within *Ctena orbiculata* (Montagu, 1808) living sympatrically with at least five other lucinid species in carbonate tidal flat sediment at Sammy Creek Landing, Sugarloaf Key, FL (USA). We selected *C. orbiculata* as the main study organism because it dominated the community. The primary goal was to investigate possible influences of environmental and spatial factors, such as occupation of seagrass- or alga-covered sediment, on endosymbiont diversity, host-symbiont functions, and strain-level diversity by comparing symbionts from multiple individual hosts of the same species. We used *C. orbiculata* gill microbiomes and metagenomes to generate bacterial taxonomic profiles and metagenome-assembled genomes (MAGs) that could test the hypothesis that strain-level *C. orbiculata* endosymbiont diversity exists within a population. Prior research had not been at a sufficiently high genetic resolution to uncover this level of endosymbiont diversity. We also used metatranscriptomic analyses to infer host-symbiont gene expression and to identify differentially expressed genes across *C. orbiculata* endosymbiont taxa and the environment.

## RESULTS

### Site characterization.

**FIG 1:**
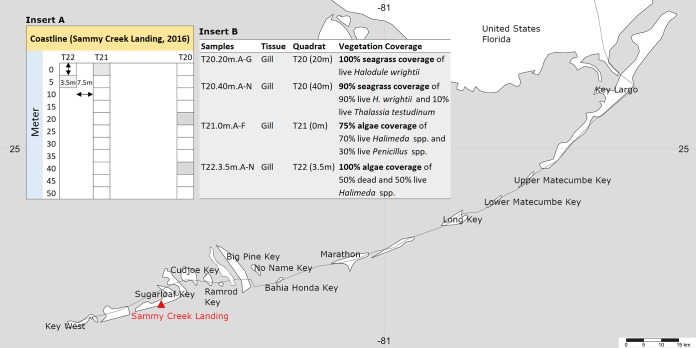
Map showing location of the sampling site at Sammy Creek Landing in Florida, USA. *Ctena orbiculata* analyses focused on specimens collected from four quadrats (shaded cells in insert A: T20 20 m and 40 m, T21 0 m, and T22 3.5 m) that were covered predominantly with either seagrass or algae (insert B). Map is from SimpleMappr (92).

A volume of nearly 3 m^3^ of carbonate sediment was hand dug and hand sieved from 13 quadrats covered with either seagrass or algae (Fig. 1; see also Table S1 in the supplemental material) to reveal a diverse lucinid community that averaged 45.5 live lucinids per m^3^, predominately from the top 30 cm of sediment. We observed an average of 46.4 live *C. orbiculata* specimens from the site quadrats (*n* = 13), 22.3 live *Codakia orbicularis* specimens, 6.2 live Lucinisca nassula specimens, and 4.1 live *Anodontia alba* specimens (Table S1). A few live individuals of Parvilucina pectinella (1.5) and Radiolucina amianta (0.8) were also recovered at the site. Lucinids were encountered from sediment that had a polymodal grain size distribution of very poorly sorted to poorly sorted coarse sand to gravelly sand with an average of 3.3% organic carbon content, regardless of sediment depth. Seagrass species encountered in the quadrats included Halodule wrightii (shoal grass), Syringodium filiforme (manatee grass), and Thalassia testudinum (turtle grass), as well as rhizophytic calcareous algae, such as *Penicillus* spp., *Halimeda* spp., other green algae, and the red algae *Goniolithon* spp. (Table S1). Porewater dissolved sulfide concentrations ranged from <30 μmol/liter to 2.9 mmol/liter, and dissolved oxygen concentrations were lower (≤31 μmol/liter) than ocean water samples for all sampled quadrats (Table S1). Dissolved methane concentrations were generally higher near the shoreline, with the highest concentration being 2.18 μmol/liter from the same quadrat as the highest sulfide measurement.

10.1128/mSystems.00280-19.8 TABLE S1 Environmental data from Sammy Creek Landing, Sugarloaf Key, FL. The estimated density of live clams was calculated from the number of live clams recovered from the volume of sediment excavated per quadrat. Dissolved sulfide and dissolved oxygen (DO) measurements are reported in milligrams per liter, dissolved methane concentrations are in micrograms per liter, temperature (temp) is in Celsius, and conductivity (cond) is in millisiemens per centimeter. Download Table S1, DOCX file, 0.01 MB. Copyright © 2019 Lim et al. 2019 Lim et al. This content is distributed under the terms of the Creative Commons Attribution 4.0 International license.

### Gill microbiome diversity.

**FIG 2:**
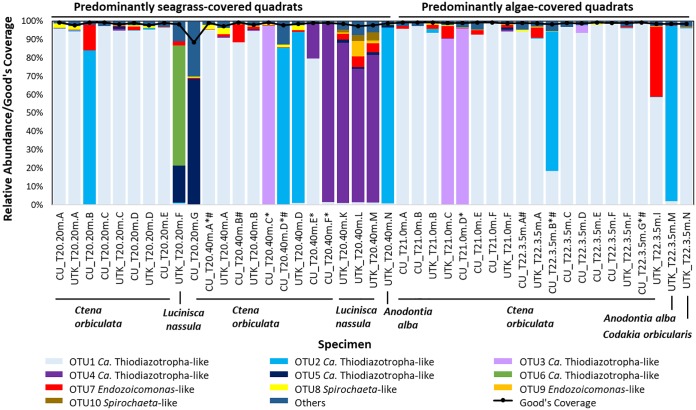
Relative abundances and Good’s coverage of subsampled bacterial OTUs identified in lucinid gill specimens collected from Sammy Creek Landing, FL, USA. CU and UTK indicate specimens sequenced by Clemson University and University of Knoxville—Tennessee groups, respectively. Asterisks denote specimens also used for metagenomic sequencing, and pound signs denote specimens used for metatranscriptomic sequencing.

From the 13 quadrats, four were chosen for detailed examination of lucinid and symbiont diversity based on seagrass or alga coverage (Fig. 1); two quadrats were dominated by Halodule wrightii and two others by *Halimeda* spp. To our knowledge, a potential association between lucinids and calcareous green algae has not been established previously. The V4 region of the 16S rRNA gene was sequenced from a total of 36 *C. orbiculata* gill samples retrieved from the four quadrats, as well as from three *L. nassula* gill samples, two *A. alba* gill samples, and one *Codakia orbicularis* gill sample. Of these hosts, 10 *Ctena orbiculata* gill samples were dissected from the same bivalves but processed and sequenced at two different institutions (see Materials and Methods), thus serving as technical replicates (Fig. 2). Sequences were clustered into operational taxonomic units (OTUs) at 99% sequence identity (26) and showed, on average, 98% ± 2% Good’s sequencing coverage (27). Taxonomic classification of resulting OTUs against the Silva v132 (28) database at 90% bootstrap confidence revealed six coexisting “*Candidatus* Thiodiazotropha”-like OTUs (OTU1 to OTU6) present at >60% relative abundances in at least one gill sample from the mixed lucinid community (Fig. 2). OTU1 dominated the gills of 18 of 26 *C. orbiculata* individuals (average relative abundance of 93% ± 8%) and a single *Codakia orbicularis* individual from the site (99% relative abundance; Fig. 2). The representative sequence for OTU1 was also 100% identical to sequences from two *Codakia orbicularis* individuals previously found in the Bahamas (Fig. S1) (7). OTU2 was predominant in the gills of three *Ctena orbiculata* individuals at 85% ± 7% average relative abundance and two *A. alba* individuals at 96% ± 0.3% average relative abundance, while OTU3 dominated the gills of three *C. orbiculata* individuals at 95% ± 4% average relative abundance (Fig. 2). The OTU3 representative sequence matched the previously sequenced V4 region of the 16S rRNA gene from *L. nassula* off Guadeloupe (22) (Fig. S1). OTU4 dominated the gills of three of four *L. nassula* individuals (80% ± 7% average relative abundance) and one *C. orbiculata* individual (97% relative abundance) but was also detected at 19% relative abundance in another *C. orbiculata* individual and 0.6% ± 0.4% relative abundance in six additional *C. orbiculata* individuals (Fig. 2). The representative sequence for OTU4 was identical to sequences retrieved from *P. pectinella* off Guadeloupe (GenBank accession no. MK346268) and *L. nassula* from Lemon Bay, FL, USA (29) (Fig. S1). OTU5 occurred at 69% relative abundance in the gills of one *C. orbiculata* individual and at 1% ± 4% average relative abundances in 17 other individuals (Fig. 2). The OTU distribution did not follow any clear spatial trend. For instance, high relative abundances of OTU1 to OTU3 were identified in gill specimens from both seagrass- and alga-covered quadrats, and OTU4 was detected at high relative abundances (>50%) in gill specimens from a seagrass-covered quadrat (T20 at 40 m) but low relative abundances in the other three quadrats (Table S1). Incidentally, as previously observed in studies on lucinid symbiont diversity (8, 30–32), *Endozoicomonas*-like (order *Oceanospirillales*) and *Spirochaeta*-like bacterial OTUs were also present in lucinid gill tissues from this site (discussed in Text S1).

10.1128/mSystems.00280-19.1 TEXT S1 Supplemental materials, methods, results, and discussion. Download Text S1, DOCX file, 0.04 MB. Copyright © 2019 Lim et al. 2019 Lim et al. This content is distributed under the terms of the Creative Commons Attribution 4.0 International license.

10.1128/mSystems.00280-19.2 FIG S1 Bootstrap consensus tree of the 10 most abundant 16S rRNA gene OTUs and 16S rRNA gene sequences recovered from metagenome-assembled genomes (MAGs) discovered in this study (red font), in relation to symbionts of other lucinid species (blue font), marine species, insect species, and free-living bacterial species. GenBank accession numbers are indicated in brackets, and the three clades comprising thioautotrophic lucinid symbionts are labeled and colored. The outgroup used was Desulfurobacterium thermolithotrophum from phylum *Aquificae*. Black circles on each node are sized proportionally to bootstrap values. Download FIG S1, PDF file, 1.6 MB. Copyright © 2019 Lim et al. 2019 Lim et al. This content is distributed under the terms of the Creative Commons Attribution 4.0 International license.

**FIG 3:**
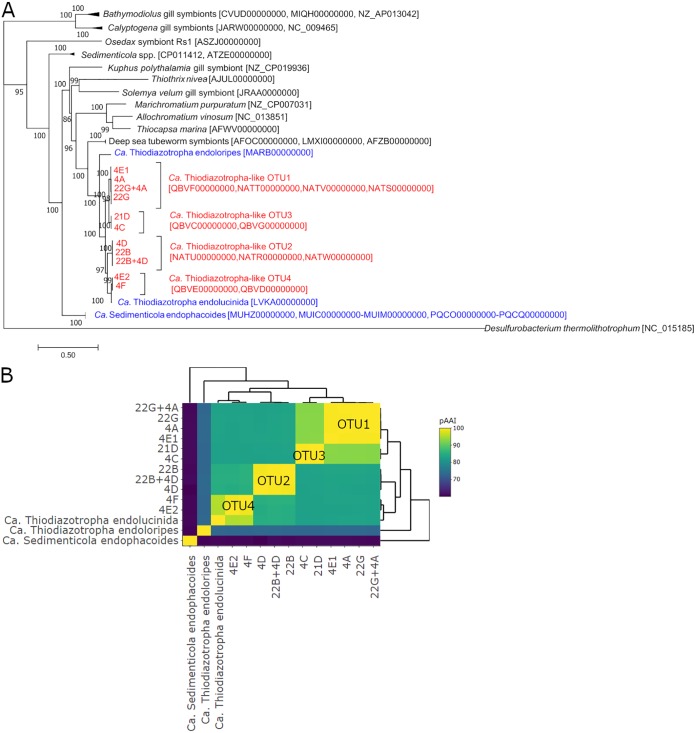
(A) Phylogenomic tree (outgroup Desulfurobacterium thermolithotrophum) of *C. orbiculata* symbiont MAGs in this study in relation to lucinids (blue), other bivalves, tubeworm symbionts, and free-living bacteria using eight single-copy marker genes (*dnaG*, *nusA*, *pgk*, *rplS*, *rpsE*, *rpsK*, *rpsM*, and *smpB*). (B) Pairwise AAI comparisons between *C. orbiculata* symbiont MAGs and other lucinid symbiont MAGs. GenBank (88) accession numbers in panel A are indicated in brackets. Tree nodes in panel A show approximate likelihood-ratio test (aLRT) SH-like support values (89), and the scale bar indicates 0.5 substitution per site.

Metagenomic sequencing of the gills from eight *C. orbiculata* specimens, which were dominated by “*Ca.* Thiodiazotropha”-like OTU1 to OTU4, yielded four OTU-specific clusters of gammaproteobacterial metagenome-assembled genomes (MAGs) (Fig. 3 and Table 1). 16S rRNA gene sequences annotated in OTU1- to OTU4-related MAGs shared 100% identity in their V4 regions with corresponding OTU sequences derived from the same gill specimens. Phylogenetic analyses using the 16S rRNA gene (Fig. S1) and eight single-copy marker genes (Fig. 3A) grouped these MAGs with other clade A thioautotrophic lucinid endosymbionts with OTU-specific clustering. MAGs within each of the four OTU-specific clusters shared >99% pairwise average nucleotide identity (pANI) and average amino identity (pAAI) with each other and were most similar to the representative MAG of “*Ca.* Thiodiazotropha endolucinida” (9) (Fig. 3B). Species classification of the MAGs, based on the 93% to 95% pANI and 85% to 90% pAAI boundaries proposed by Rodriguez-R. and Konstantinidis (33), showed that the OTU1- and OTU3-related MAGs, which shared 91% ± 0.2% pANI and 93% ± 0.06% pAAI to each other, were likely different strains of the same species (Fig. 3B). However, OTU1 and OTU3 likely represent a species separate from “*Ca.* Thiodiazotropha endolucinida” (71% ± 5% pANI; 83% ± 0.1% pAAI), OTU2 (80% ± 0.7% pANI; 83% ± 0.2% pAAI), and OTU4 (79% ± 0.8% pANI; 83% ± 0.2% pAAI) (Fig. 3B). OTU2-related MAGs and OTU4-related MAGs shared 81% ± 1% pANI and 85% ± 0.4% pAAI with each other and 75% ± 1% pANI and 85% ± 0.2% pAAI, and 73% ± 1% pANI and 95% ± 0.08% pAAI, respectively, with the representative “*Ca.* Thiodiazotropha endolucinida” MAG (Fig. 3B). Therefore, OTU2, OTU4, and “*Ca.* Thiodiazotropha endolucinida” could be considered different strains of the same species.

**TABLE 1:** Features of MAGs recovered from *C. orbiculata* gill specimens^*a*^

Categorized species/strain and quadrat	MAG(s)	No. of PE reads (million)	Size (Mb)	No. of contigs	No. of PEGs	% G+C	*N*_50_ (kb)	Completeness by method:		% contamination	% strain heterogeneity	MAG quality (8)
								CheckM (9)	BUSCO (10)			
OTU1												
T22 (3.5 m)	22G	0.5	4.0	235	3,718	56	26	97	94	2	0	High
T20 (40 m)	4A	0.5	3.9	350	3,638	56	16	94	89	2	0	High
Mixed	22G + 4A	1	4.3	182	3,944	56	36	98	96	2	17	High
OTU3												
T20 (40 m)	4E1	5.8	4.2	122	3,833	56	50	98	96	3	33	High
T20 (40 m)	4C	6.2	4.6	42	4,070	57	211	98	94	2	20	High
T21 (0 m)	21D	5.1	4.5	51	4,049	58	139	98	95	2	0	High
OTU2												
T22 (3.5 m)	22B	0.3	4.0	309	3,700	54	19	97	88	4	28	Medium
T20 (40 m)	4D	0.5	3.4	656	3,127	53	6	84	69	2	0	Medium
Mixed	22B + 4D	0.8	4.1	193	3,729	53	34	98	96	3	8	Medium
OTU4												
T20 (40 m)	4E2	5.8	4	593	3,730	53	9	91	77	4	37	Medium

a Abbreviations: PE, paired end; PEGs, protein-encoding genes.

**FIG 4:**
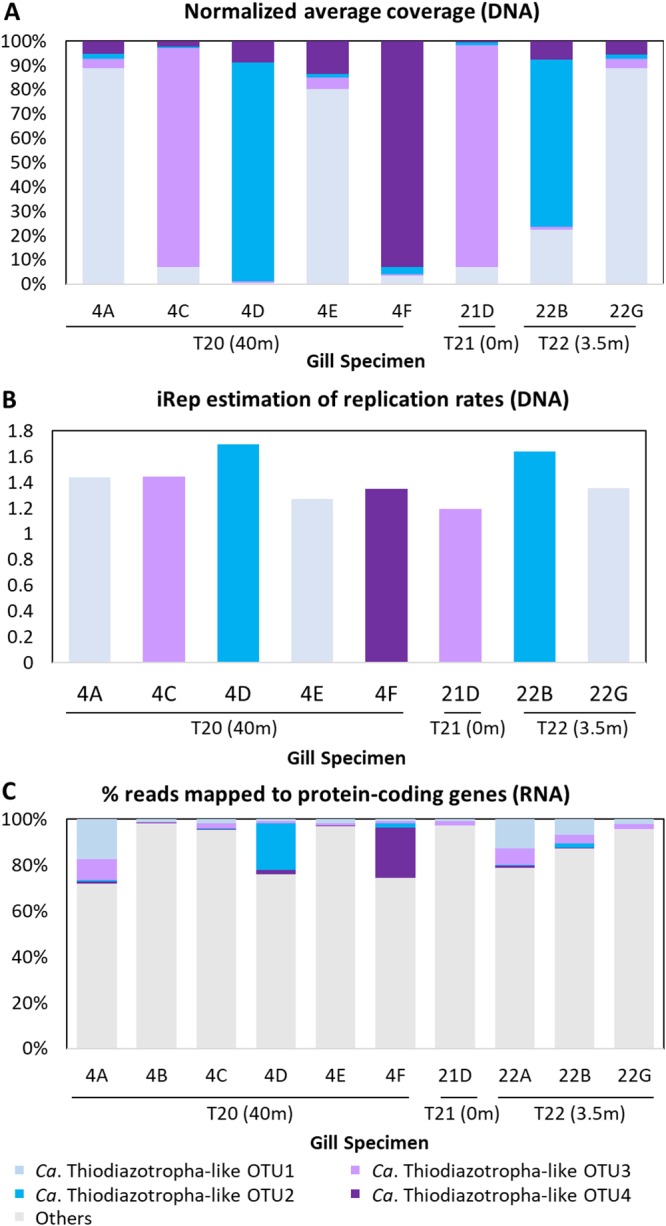
(A) Percent average coverage depths normalized by MAG size. (B) iRep (34) estimation of replication rates. (C) Percent metatranscriptomic reads of each sequenced gill specimen from specific quadrats mapped to each representative taxon-specific MAG. Only bars with an ≥0 estimated replication rate are shown in panel B.

**FIG 5:**
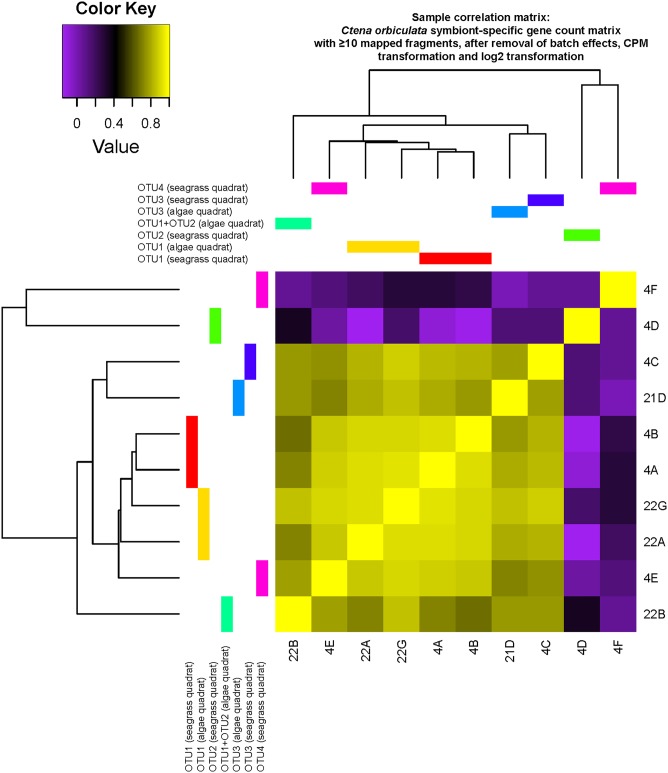
Heat map of pairwise Pearson correlations across gill specimens, based on the number of assembled transcripts mapped to genes in symbiont transcriptomes extracted from the metatranscriptomic assembly. The count matrix was processed to filter out genes with <10 mapped fragments and to eliminate batch effects and was normalized to log_2_ counts per million (CPM).

Metagenomic read coverage profiles of each representative OTU-specific MAG, bacterial replication rates estimated by iRep (34) from MAG data, and percentages of metatranscriptomic reads mapped to protein-encoding genes of each representative OTU-specific MAG were generally consistent with relative abundance patterns of their corresponding 16S rRNA gene OTU (Fig. 4). The only exception was OTU2-dominated gill metatranscriptome 22B from quadrat T22 (3.5 m), which showed higher percentages of reads mapped to OTU1 than to OTU2 (Fig. 4C). Symbiont-specific gill transcriptomes of OTU1-/OTU3-dominated specimens clustered together with an 0.9 ± 0.07 average pairwise Pearson correlation coefficient (PCC), while the OTU2-dominated symbiont transcriptome 4D from quadrat T20 (40 m) and OTU4-dominated symbiont transcriptome 4F from quadrat T20 (40 m) appeared to be outliers sharing <0.4 PCC with the other specimens (Fig. 5). Symbiont OTU-specific patterns were not observed across the entire gill metatranscriptomes (average 0.7 ± 0.4 PCC between samples) or another subset of Mollusca-related transcriptomes (average 0.8 ± 0.06 between samples). Host 18S rRNA, 28S rRNA, and mitochondrial cytochrome *b* (*cytb*) gene sequences extracted from unbinned *C. orbiculata* gill metagenomes clustered unambiguously with reference sequences from *C. orbiculata* (Fig. S2), thereby confirming the host taxonomy of these specimens.

10.1128/mSystems.00280-19.3 FIG S2 Maximum likelihood tree of 18S rRNA gene and 28S rRNA gene sequences (A) and cytochrome *b* gene sequences (B) from *C. orbiculata* in relation to reference lucinid species. Tree nodes show bootstrap values, and brackets contain GenBank accession numbers for reference sequences. *Thyasira polygona* (order Lucinida, family Thyasiridae) was used as the outgroup in both trees. Scale bars indicate the number of substitutions per site. Download FIG S2, TIF file, 0.8 MB. Copyright © 2019 Lim et al. 2019 Lim et al. This content is distributed under the terms of the Creative Commons Attribution 4.0 International license.

### Core symbiont functions.

Pangenomes of “*Ca.* Thiodiazotropha”-like MAGs were predicted by Rapid Annotation using Subsystem Technology (RAST) (35) to share ∼62% gene and ∼83% subsystem content. As noted by Lim et al. (8), gene and subsystem annotations were based on incompletely sequenced and annotated MAGs. As such, the numbers of shared genes and subsystems presented here are imprecise estimates that cannot account for missing, unbinned, or unclassifiable genes or for incomplete pathways and potential strain/cross-species contamination of the MAGs. Moreover, limitations of host-symbiont metatranscriptomic analyses also apply (8).

**FIG 6:**
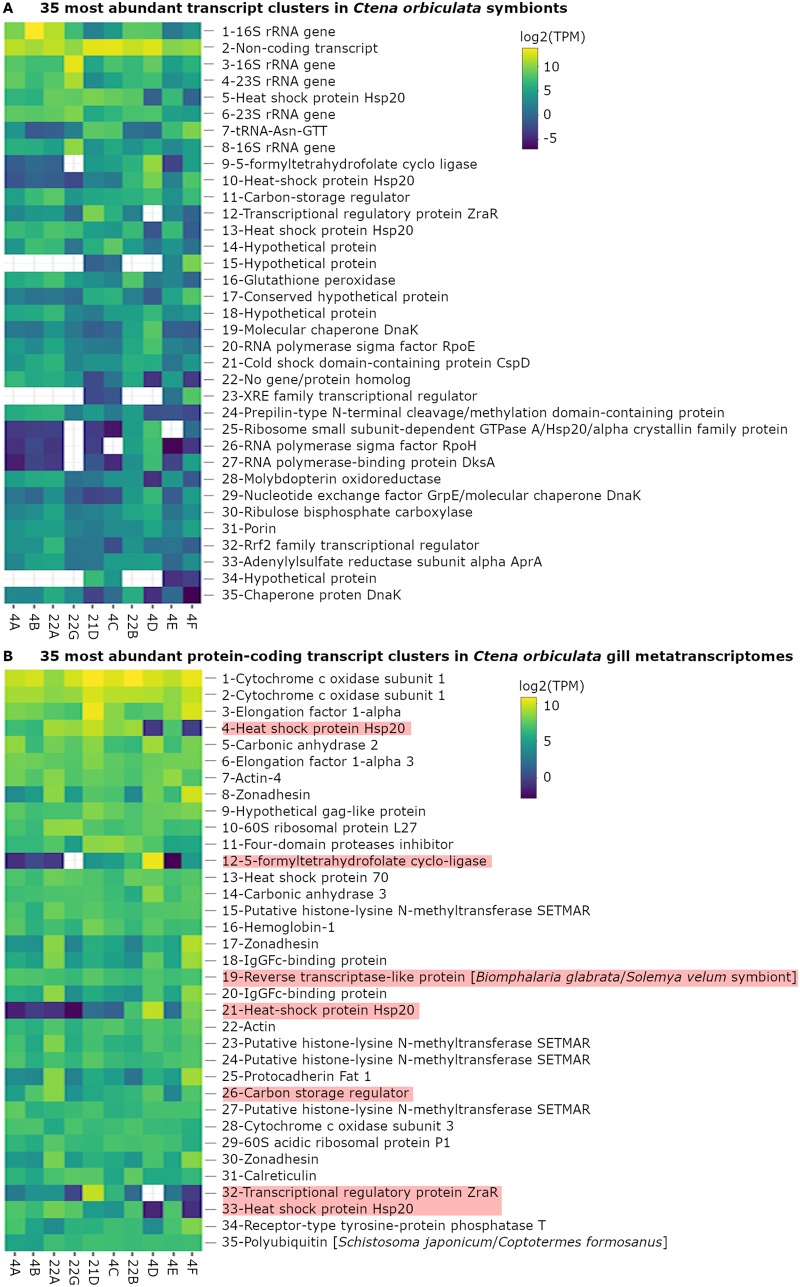
Log_2_-transformed trimmed mean of M-values (TMM)-normalized TPM of gene products of the 35 most abundantly expressed transcript clusters mapped to *C. orbiculata* symbionts (A) and protein-encoding transcript clusters in sequenced gill metatranscriptomes (B). Bacterium-related transcripts in panel B are highlighted in pink.

Nevertheless, even with incompletely annotated MAGs, the *C. orbiculata* symbionts showed high expression of a carbon storage regulator (103 ± 97 average trimmed mean of M-value [TMM] normalized transcripts per million [TPM]), thioautotrophy-related form Iaq RuBisCO (average 18 ± 19 TPM), and adenylsulfate reductase subunit A (average 15 ± 12 TPM), which were among the 35 most abundant transcript clusters (loosely equivalent to genes) in the symbiont transcriptomes (Fig. 6). Specifically, MAGs of OTU1, OTU3, and “*Ca.* Thiodiazotropha endoloripes” (7) encoded and expressed form Iaq RuBisCO, whereas OTU2, OTU4, and “*Ca.* Thiodiazotropha endolucinida” (9) encoded and expressed form Iaq and form II RuBisCO (Fig. S3). Form II RuBisCO was expressed only in the OTU2-dominated gill specimen 4D from the seagrass quadrat T20 (40 m) at 0.205 TPM and was not identified in the unbinned metagenomes of OTU1- and OTU4-dominated gill specimens. For contrast, only form II RuBisCO was predicted in the mangrove-associated *P. pectinatus* symbiont species, “*Candidatus* Sedimenticola endophacoides” (8). From the *C. orbiculata* MAGs, many stress-related symbiont transcript clusters encoding multiple heat shock proteins (average 164 ± 220 TPM), the antioxidant glutathione peroxidase (average 66 ± 93 TPM), envelope stress-associated RNA polymerase sigma factor RpoE (average 37 ± 67 TPM) (36), a cold shock domain-containing protein (average 34 ± 31 TPM), heat stress-associated RpoH (37), and the stringent response-associated RNA polymerase binding protein DksA (38) were abundant (Fig. 6). In addition to thioautotrophy and mixotrophy, other functions common to the *C. orbiculata* symbiont species and other previously characterized thioautotrophic lucinid symbiont species (7–9) included hydrogenotrophy (average 1 ± 2 TPM), ammonia uptake (average 0.4 ± 0.7 TPM), denitrification (average 0.5 ± 1 TPM), assimilatory nitrate reduction (average 0.03 ± 0.04 TPM), and diazotrophy (average 0.08 ± 0.1 TPM) (Fig. S3a). Genetic evidence for urea hydrolysis in *C. orbiculata* symbionts was weak (Text S1). The *C. orbiculata* symbionts expressed flagellum-related genes (average 0.9 ± 5 TPM) and pilus-related genes (average 1 ± 7 TPM), as well as genes associated with phosphate uptake (average 0.3 ± 0.8 TPM), polyphosphate utilization (average 0.2 ± 0.6 TPM), and iron uptake (average 0.2 ± 0.4 TPM). Biosynthetic genes for all 20 essential amino acids and vitamins B_1_, B_2_, B_6_, B_7_, and B_9_ and type I, II, and VI secretion system genes were also identified in symbiont MAGs and transcriptomes. *Ctena orbiculata* symbionts also encoded and expressed genes for the valine-glycine repeat protein G (VgrG; average 0.7 ± 1 TPM) but not hemolysin-coregulated protein (Hcp) and VasL, which is exclusive to the type VI secretion system 2 gene cluster (39).

10.1128/mSystems.00280-19.4 FIG S3 (A) Key hydrogen (purple), sulfur (red), nitrogen (green), and carbon (orange) pathways shared among pangenomes of *C. orbiculata* symbionts. (B) Comparison of the Calvin-Benson-Bassham (*cbb*) operon structures in *C. orbiculata* and other thioautotrophic lucinid symbiont species. Black arrows in panel B depict genes encoding hypothetical proteins, colored arrows depict genes conserved in at least two species, and white arrows depict nonconserved genes. Abbreviations: S^0^, elemental sulfur; Fcc, flavocytochrome c, sulfide dehydrogenase; Sox, sulfur oxidation gene cluster; Sqr, sulfide:quinone oxidoreductases; Dsr, reverse dissimilatory sulfite reductase; Apr, adenylylsulfate reductase; APS, adenosine-5′-phosphosulfate; Sat, sulfate adenylyltransferase; GS, glutamine synthetase; Fd-GOGAT, ferrodoxin-dependent glutamate synthase; GOGAT, glutamine oxoglutarate aminotransferase (glutamate synthase); ABC, ATP-binding cassette transporters; Nas, assimilatory nitrate reductase; Nit, assimilatory nitrite reductase; Nif, nitrogen fixation gene cluster; Nap, periplasmic dissimilatory nitrate reductase; Nir, cytochrome nitrite reductase cd1; Nor, nitric oxide reductase; Nos, nitrous oxide reductase; ActP, acetate permease; TRAP, tripartite ATP-independent periplasmic transport; RuBisCO, ribulose-1,5-bisphosphate carboxylase/oxygenase; Mdh, pyrroloquinoline-quinone (PQQ)-dependent methanol dehydrogenase; Fdh, NAD-dependent tungsten-containing formate dehydrogenase; Fdo, formate dehydrogenase O; IM, inner membrane; OM, outer membrane. Download FIG S3, TIF file, 2.8 MB. Copyright © 2019 Lim et al. 2019 Lim et al. This content is distributed under the terms of the Creative Commons Attribution 4.0 International license.

**FIG 7:**
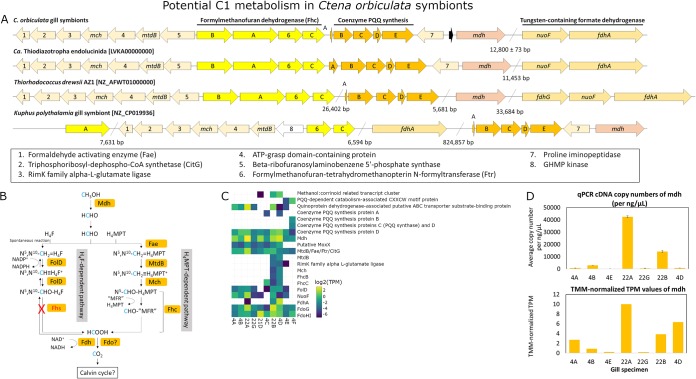
(A) Conserved gene clusters. (B) Proposed pathways modified from references 90 and 91. (C) TMM-normalized log_2_ TPM. (D) qPCR copy numbers and TMM-normalized TPM values of methanol dehydrogenase and/or other C_1_ oxidation genes in *Ctena orbiculata* symbiont species. Colored arrows in panel A depict genes conserved in at least two species, and the black and white arrows represent genes encoding hypothetical and nonconserved proteins, respectively. Abbreviations: Mch, methenyl-tetrahydromethanopterin cyclohydrase; MtdB, NAD(P)-dependent methylene-tetrahydromethanopterin dehydrogenase; Mdh, pyrroloquinoline-quinone (PQQ)-dependent methanol dehydrogenase; NuoF, NADH-quinone oxidoreductase subunit F; Fdh, NAD-dependent tungsten-containing formate dehydrogenase; RimK, ribosomal protein S6 modification enzyme; GHMP, galacto-, homoserine, mevalonate, and phosphomevalonate; H_4_F, tetrahydrofolate; FolD, bifunctional methylene-H_4_F dehydrogenase/methenyltetrahydrofolate cyclohydrolase; H_4_MPT, tetrahydromethanopterin; Fhc, formyltransferase/hydrolase complex; Fdo, formate dehydrogenase O; Fae, formaldehyde activating enzyme; “MFR,” postulated methanofuran analogue; Fhs, formyl-H_4_F synthetase; ABC, ATP-binding cassette; MoxX, methanol utilization control regulatory protein.

In addition to thiotrophy and hydrogenotrophy-related genes, a C_1_ oxidation gene cluster encoding proteins involved in pyrroloquinoline quinone (PQQ) synthesis (average 0.2 ± 0.6 TPM), PQQ-dependent methanol oxidation (Mdh; average 3 ± 3 TPM), and tetrahydromethanopterin (H_4_MPT)-dependent formaldehyde oxidation (average 0.2 ± 0.6 TPM) was conserved in all sequenced *C. orbiculata* symbionts and “*Ca.* Thiodiazotropha endolucinida” (8) (Fig. 7). Downstream of this gene cluster, another formate oxidation gene cluster encoding NADH-quinone oxidoreductase subunit F and formate dehydrogenase alpha subunit (FdhA) was predicted in all *C. orbiculata* symbionts, “*Ca.* Thiodiazotropha endolucinida” (9), and “*Ca.* Thiodiazotropha endoloripes” (7). Phylogenetic analyses of Mdh and FdhA protein sequences showed OTU-specific clustering patterns across *C. orbiculata* symbionts (Fig. S4 and S5 and Text S1) consistent with the 16S rRNA gene tree (Fig. S1) and phylogenomic tree (Fig. 3A). These sequences were most closely related to each other and more distantly associated with the giant Teredinidae bivalve Kuphus polythalamia (40) and other free-living bacterial species (Text S1). Two sets of qPCR primers targeting Mdh from OTU1 and OTU2 were designed to amplify DNA and cDNA from gill specimens (Table S2), and the qPCR cDNA copy numbers of Mdh were generally consistent with TPM values observed in five out of seven amplified gill specimens (Fig. 7D). Other genes potentially related to C_1_ oxidation were also annotated in MAGs and/or transcriptomes of *C. orbiculata* symbionts (Text S1). C_1_ assimilation genes in the ribulose monophosphate (RuMP) pathway, and four out of 10 key genes (serine hydroxymethyltransferase, malyl coenzyme A lyase [malyl-CoA-lyase], malate dehydrogenase, and enolase) in the serine-glyoxylate cycle (41), were identified in the symbiont MAGs. These four genes, as well as 11 additional accessory genes assigned by RAST (35) to the serine-glyoxylate cycle subsystem, were also predicted in other carbon-related pathways, including biosynthesis, glycolysis, photorespiration, tricarboxylic acid cycle, glyoxylate cycle, polyhydroxybutyrate metabolism, acetyl-CoA fermentation to butyrate, and phenylalkanoic acid degradation.

10.1128/mSystems.00280-19.5 FIG S4 Unrooted bootstrap consensus maximum likelihood tree of methanol dehydrogenase protein sequences from *C. orbiculata* (red) in relation to other lucinid symbionts (blue) and other bacterial species. Tree nodes show bootstrap values, and brackets contain GenBank accession numbers. Download FIG S4, PDF file, 0.8 MB. Copyright © 2019 Lim et al. 2019 Lim et al. This content is distributed under the terms of the Creative Commons Attribution 4.0 International license.

10.1128/mSystems.00280-19.9 TABLE S2 List of PCR and qPCR primers used in this study. Download Table S2, DOCX file, 0.01 MB. Copyright © 2019 Lim et al. 2019 Lim et al. This content is distributed under the terms of the Creative Commons Attribution 4.0 International license.

### Core host functions.

Gill metagenomic and metatranscriptomic data also provided information on highly expressed host functions. Carbonic anhydrase transcript clusters mapped to molluscan species (average 231 ± 149 TPM) and the sea lamprey Petromyzon marinus (average 140 ± 86 TPM), together with a lucinid-associated hemoglobin 1 transcript cluster (average 124 ± 60 TPM), were among the 20th most abundant protein-encoding transcripts in the gill metatranscriptomes, respectively (Fig. 6B). In contrast to hemoglobin 1, lucinid-associated hemoglobins 2 (average 0.2 ± 0.7 TPM) and 3 (average 0.8 ± 2 TPM) were expressed at much lower levels. Two Mollusca-related transcript clusters encoding IgG Fc-binding proteins (average 117 ± 144 TPM) were also among the 25th most abundantly expressed in the gills (Fig. 6B). This has not yet been observed in other lucinid species but may contribute to mucosal defense (42) or facilitate initial symbiont selection of tightly aggregated symbionts as in the *Euprymna*-*Vibrio* symbiosis (43). Among lysozyme-associated transcripts in *C. orbiculata* (average 0.9 ± 2 TPM), presumably expressed to enable microbiome selection and/or digestion (8, 44), one molluscan-related transcript cluster encoding lysozyme 3 was the most highly expressed (average 4 ± 2 TPM). Bivalve-related transcript clusters homologous to urease (average 1 ± 3 TPM), urease accessory proteins (average 0.2 ± 0.2 TPM), and urease transporters (average 0.2 ± 0.5 TPM) were also identified in all gill metatranscriptomes.

### Symbiont species/strain differences.

In addition to interspecies differences in the forms of RuBisCO identified, *C. orbiculata* symbiont MAGs and transcriptomes showed very little interstrain and interspecies variation of complete or nearly complete metabolic pathways. For aerobic respiration, *C. orbiculata* symbionts and other clade A lucinid symbionts (7, 9) potentially utilize cbb3 (average 4 ± 5 TPM in the former) and aa3 terminal oxidases (average 5 ± 9 TPM). Furthermore, OTU2-related MAGs and transcriptomes contained genes for cytochrome *bd* ubiquinol oxidase (average 0.5 ± 0.4 TPM) that were also detected in the unbinned metagenomic assembly and metatranscriptome of OTU4-dominated gill specimen 4F from the seagrass-covered quadrat T20 (40 m) at 0.02 TPM, as well as the metatranscriptome of OTU3-dominated gill specimen 4C from quadrat T20 (40 m) at 0.3 TPM. *Ctena orbiculata* symbionts also likely use distinct types of clustered regularly interspaced short palindromic repeats (CRISPR)-associated genes (Text S1).

Differential expression (DE) analyses on symbiont-related genes using four DE algorithms showed <20 total DE transcript clusters (*P* < 0.05, ≥2-fold change) for OTU2-OTU3, OTU2-OTU4, and OTU3-OTU4 community comparisons (Fig. S6). On the other hand, OTU1-OTU3 comparisons showed four total upregulated and 88 downregulated genes in OTU1-dominated communities, whereas OTU1-OTU2 comparisons showed 751 total upregulated and 403 downregulated genes in OTU1-dominated communities (Fig. S6). OTU1-OTU4 comparisons showed 10 upregulated and 72 downregulated genes in OTU1-dominated communities (Fig. S6). These genes were mainly involved in secretion, stress response, transport, and biosynthesis functions (Text S1).

10.1128/mSystems.00280-19.6 FIG S5 Unrooted bootstrap consensus maximum likelihood tree of formate dehydrogenase alpha protein sequences from *C. orbiculata* (red) in relation to other lucinid symbionts (blue) and other bacterial species. Tree nodes show bootstrap values, and brackets contain GenBank accession numbers. Download FIG S5, TIF file, 0.9 MB. Copyright © 2019 Lim et al. 2019 Lim et al. This content is distributed under the terms of the Creative Commons Attribution 4.0 International license.

10.1128/mSystems.00280-19.7 FIG S6 Venn diagrams of the numbers of differentially expressed (DE) genes (*P* < 0.05, fold change of ≥2) predicted by four different algorithms across *C. orbiculata* symbiont taxa. Boxes show enriched gene ontology (GO) terms (*P* < 0.05) within categories containing >20 total DE genes. Download FIG S6, TIF file, 0.9 MB. Copyright © 2019 Lim et al. 2019 Lim et al. This content is distributed under the terms of the Creative Commons Attribution 4.0 International license.

### DE analyses of host-symbiont gene expression across quadrats.

Besides taxon-specific clustering patterns that were evident from 16S rRNA gene profiles and sequence content of the MAGs, OTU1-dominated symbiont-specific transcriptomes formed two separate subclusters based on whether specimens were collected from alga-covered and seagrass-covered quadrats (Fig. 2 and 5), although these transcriptomes shared 0.9 ± 0.01 average pairwise PCC with each other. Exploratory DE analyses on these transcriptomes showed only five symbiont-related DE genes predicted by one (DESeq2) (45) of four DE analysis algorithms used. Of these, the dissimilatory sulfite reductase transferase protein DsrC was upregulated in the alga-covered quadrat, but three transcript clusters that were upregulated in the seagrass-covered quadrat encoded hypothetical proteins and contained one transcript cluster homologous to a cell wall-associated hydrolase from an alphaproteobacterium. Host-related transcripts did not exhibit the same clustering patterns between alga- and seagrass-covered quadrats but instead showed 73 putative DE genes predicted by both DESeq2 (45) and edgeR (46) across OTU1-dominated metatranscriptomes in these quadrats. According to UniProt (47) annotations, these host-related DE genes were involved in a variety of muscle-related, cytoskeletal, cochaperone, transcriptional, translational, and other functions. Notably, host-related transcripts encoding cytochrome *b*-*c*1 complex, mitochondrial succinate dehydrogenase, and mitochondrial sulfide:quinone oxidoreductase (Sqr) were predicted to be upregulated if originating from seagrass-covered quadrats.

## DISCUSSION

To date, the taxonomic and functional diversities of lucinid symbiont species remain largely unexplored because, out of >100 identified lucinid species listed in the NCBI database (48), only three gill endosymbionts, each from a different host species, have been comprehensively sequenced (7–9). In spite of previous marker gene-based diversity studies (20, 22, 23, 25), and the recently characterized gill microbiome, metagenome, and metatranscriptome from “*Ca*. Sedimenticola endophacoides” from *P. pectinatus* (8), in-depth investigations into symbiont variation within a single lucinid host population are currently lacking. Our evidence for endosymbiont species- and strain-level taxonomic, genetic, and functional diversity within a single lucinid host expands previously reported findings of strain-level diversity within a single thioautotrophic endosymbiont species (25). Similar functionally homogenous but taxonomically heterogenous marine symbiont communities have been reported, for example, in the light organs of the squids Sepiola affinis and Sepiola robusta, where closely related Vibrio fischeri and Vibrio logei were detected at different abundances (49). The *C. orbiculata* gill metatranscriptomic analyses further reveal differentially expressed host and/or endosymbiont genes of different bacterial taxa. Although gill endosymbiont OTUs from *C. orbiculata* shared a high number of core genes and functions, which included a well-conserved and previously undiscovered C_1_ oxidation pathway, the discovery of other genetic and functional differences among coexisting “*Ca.* Thiodiazotropha”-like endosymbiont taxa supports our hypothesis that strain- and even species-level endosymbiont diversity can exist within a single host population living under variable environmental conditions. Moreover, because we also identified “*Ca.* Thiodiazotropha”-like OTUs among other sympatric lucinid species, genetic and functional trait similarities with *C. orbiculata* endosymbionts likely exist within other lucinid populations, which emphasizes the importance of future investigations of these chemolithoautotrophic symbiotic systems at broader population, community, and ecosystem levels.

The thioautotrophic *C. orbiculata* endosymbionts shared a high number of common genes and functions, including previously characterized lithoautotrophy, diazotrophy, and potential heterotrophy (7–9), as well as the oxidation of C_1_ compounds, including methanol, formaldehyde, and formate. These potential C_1_ compound oxidation functions were also discovered in MAGs of “*Ca.* Thiodiazotropha endolucinida” (9) that had been unrecognized and undescribed until our analyses. *Ctena orbiculata* endosymbionts may use C_1_ compounds as an energy source because they encoded and expressed formate dehydrogenase O that enables the use of formate as an electron donor during respiration (50). Because there was no substantial genetic evidence for RuMP and serine-glyoxylate C_1_ assimilation pathways, we hypothesize that the CO_2_ end product of C_1_ oxidation for these endosymbionts could be fixed via the autotrophic Calvin-Benson-Bassham cycle, as demonstrated in the diazotrophic alphaproteobacterial *Xanthobacter* strain 25a (51). Additionally, many transcripts involved in temperature, oxidative, envelope, and nutritional stress responses were highly expressed in *C. orbiculata* endosymbionts and likely reflect stresses caused by the external environment or stresses due to intracellular host selection mechanisms (8), the latter being similar to the *Euprymna*-*Vibrio* symbiotic system (52–54). Defense-related transcripts involved in type I, II, and VI secretion systems, such as colicin-related transcripts, general secretion (Sec)-related transcripts, and twin-arginine translocation (Tat)-related transcripts, may reduce symbiont-symbiont competition by killing closely related strains (8, 55), as well as contributing to host infection (56, 57). Also, differentially expressed genes for secretion, stress response, transport, and biosynthesis proteins across the *C. orbiculata* endosymbiont taxa may also be due to variable habitat conditions.

For aerobic respiration, *C. orbiculata* endosymbionts and other seagrass-associated lucinid symbionts (7, 9) can potentially use the high-affinity cbb3-type and low-affinity aa3-type terminal oxidases under low and high oxygen concentrations (58, 59), respectively. Additionally, cytochrome *bd* ubiquinol oxidase, adapted to microaerobic environments (60), was encoded and expressed mainly by the “*Ca.* Thiodiazotropha”-like OTU2. This enzyme may facilitate symbiotic nitrogen fixation via oxygen scavenging and respiratory protection, as demonstrated in other free-living and plant-associated nitrogen-fixing bacteria (61–63), along with glutathione peroxidase that confers similar protective functions by scavenging H_2_O_2_, as shown in the legume-*Rhizobium* symbiosis (64). Conversely, RuBisCO form Iaq, detected in “*Ca.* Thiodiazotropha”-like OTU1 to OT4, is more efficient than form II, detected in OTU2 and OTU4, at distinguishing between the competing substrates oxygen and CO_2_ (65). Differences in aerobic oxidase and RuBisCO forms, as well as highly expressed antioxidant glutathione peroxidase transcripts, indicate that the symbionts experience higher or variable levels of oxygen in their environments, which could be during their intracellular free-living or extracellular host-associated growth stages. Analyses of host-related transcripts revealed 100-fold-higher expression of sulfide-reactive hemoglobin 1 (66) in *C. orbiculata* compared to oxygen-reactive hemoglobins 2 and 3 (66). This is in contrast with previous observations in *P. pectinatus*, where consistently similar high expression levels of hemoglobins 1, 2, and 3 were detected (8). Compared to alga-covered quadrats, *C. orbiculata* host-related transcripts in seagrass-covered quadrats showed candidate upregulated genes that encoded cytochrome *b*-*c*1 complex involved in aerobic respiration and oxidative stress-triggered apoptosis (67), mitochondrial succinate dehydrogenase that connects the tricarboxylic acid cycle to the electron transport chain (68), and mitochondrial Sqr that oxidizes sulfide to thiosulfate (69). Collectively, these findings indicate that complex host-symbiont cooperative regulation of intracellular oxygen and sulfide levels facilitates thioautodiazotrophic processes, which are distinct from the *P. pectinatus* chemolithoautotrophic symbiotic system (8).

Unlike previous studies on Caribbean lucinid symbiont species (25) and *Sepiola* squid symbioses (70), where species distribution is influenced by host geography and temperature, respectively, we did not uncover extensive spatial controls or apparent nonrandom patterns of strain-level *C. orbiculata* endosymbiont species distribution. The observed endosymbiont diversity from *C. orbiculata* and other sympatric lucinid species could be due to constant symbiont turnover, because adult *C. imbricatula* clams (referred to as *Codakia orbiculata* in reference 21) have been reported to lose their chemosynthetic endosymbionts when starved for sulfide and reacquire endosymbionts from their natural habitat. This behavior points to continuous horizontal symbiont acquisition throughout the life span of this host species (71, 72). However, a recent cross-infection experiment demonstrates that starved *C. imbricatula* (referred to as *Codakia orbiculata* in reference 21) adults with mature bacteriocytes could reacquire only symbiont strains that they initially hosted (25). In contrast, *Codakia orbicularis* juveniles, presumably with naive bacteriocyte precursors, acquire symbionts from purified gill-symbiont sections of their own species, as well as other lucinid species hosting thioautotrophic symbionts with identical 16S rRNA gene sequences (24, 25). On the other hand, starvation-related symbiont loss has not been observed in *Lucina roquesana* (referred to as *Lucina pensylvanica* in references 21 and 73). The contrasting evidence for symbiont acquisition, loss, and reacquisition suggests that such mechanisms could be host species specific in lucinid bivalves (73). Based on our results, it does not appear that a single symbiont species occupies a single host. Instead, a single host may harbor multiple symbiont species and strains. This is likely due to either higher taxonomic symbiont diversity encountered by the host in the environment (20) or less stringent host regulation of symbiont acquisition in the mixed lucinid community (25). Interhost symbiont transmission could also be a possible reason for the observed symbiont diversity in the mixed lucinid community. However, previous studies on *C. imbricatula* (referred to as *Codakia orbiculata* in reference 21) and *Codakia orbicularis* ruled out the possibility of host-to-host symbiont transfer by showing that adults do not release their thioautotrophic symbionts into the environment (74). In addition, opportunistic host-microbe associations have been proposed for lucinid bivalves (20) and observed in the green macroalga Ulva australis (75). Therefore, symbiont acquisition by this mixed lucinid community may be based on random encounters during the juvenile stage, as well as the diversity and distribution of “*Ca*. Thiodiazotropha” species in this habitat, which are under investigation. Nevertheless, our results suggest that a taxonomically heterogenous symbiont community, rather than a monospecific, homogenous symbiont community, exists in some lucinid species.

Overall, our study uncovered remarkable taxonomic, genetic, and functional thioautotrophic gill endosymbiont diversity in *C. orbiculata* and furthers the current understanding of lucinid-symbiont association patterns, physiology, and interactions. Despite the small sample size and replication limitations that potentially affect the statistical significance of DE analysis, as well as the lack of pure symbiotic monocultures, our findings highlight the intriguing, poorly understood complexity of lucinid-bacterium chemolithoautotrophic symbioses and generate a range of new testable hypotheses to evaluate the establishment, persistence, stability, and distribution of symbiont communities within hosts and their environment. Future studies to understand symbiont taxonomy, intracellular spatial distribution, functional diversity, and host-symbiont specificity across environmental gradients should include cross-infection experiments, imaging experiments involving fluorescence *in situ* hybridization, controlled aquarium experiments, and large-scale field studies coupled with -omics analyses. This work also proposes future in-depth investigations on C_1_ metabolism in thioautotrophic lucinid symbionts and three-way interactions between the environment, lucinid hosts, and their symbionts. Additionally, targeted experimental approaches will be useful in examining similarities and differences in phenotypic effects that various symbiont taxa and lucinid hosts exert on each other. In each of the previous -omics studies of lucinid chemolithoautotrophic symbiotic systems, a unique species name for the lucinid endosymbiont has been proposed, such as the two clade A endosymbionts, “*Ca.* Thiodiazotropha endolucinida” from *Codakia orbicularis* (9) and “*Ca.* Thiodiazotropha endoloripes” from *Loripes orbiculatus* (7). For the clade C endosymbiont from the mangrove-dwelling *P. pectinatus* (8), which does not fix nitrogen and instead belongs to the genus *Sedimenticola*, the candidate name for the endosymbiont was justifiably proposed as “*Ca*. Sedimenticola endophacoides” (8). However, based on our analyses here, we think it is too premature to assign a species name for the *C. orbiculata* symbionts. Even if “*Ca.* Thiodiazotropha endolucinida” is likely appropriate for the OTU2 and OTU4 strains, the OTU1 and OTU3 strains likely belong to a different species, but this species is not specific to *C. orbiculata* or even to lucinid hosts having clade A endosymbionts. Therefore, we contend that future lucinid symbiont taxonomic assignments and classification need to be based on detailed investigations of more lucinid symbionts from all three endosymbiont clades and from different habitats.

## MATERIALS AND METHODS

### Study area and sediment characterization.

Field sampling was done on the carbonate tidal flat at Sammy Creek Landing, on Sugarloaf Key, FL (USA), from 27 June to 1 July 2016. Twelve quadrats were separated by 5 m along two 50-m-long transects (six quadrats each) that ran perpendicular to the shoreline; a 13th quadrat was established 3.5 m away from shore to accommodate equal sampling of seagrass versus benthic green and red algal cover (Fig. 1). The seagrass or algal cover was estimated for each quadrat before hand digging and hand sieving the sediment to uncover lucinids and other infauna. In general, depths of sediment excavation varied, from 0.29 to 0.5 m, and generally stopped at a very coarse, organic-material-rich sediment layer below which lucinids were not recovered. Intermixed with the sediment was abundant road (e.g., concrete and asphalt) and other anthropogenically sourced debris, such as manufactured wood, roofing material, and mixed-use plastic, likely due to past storm activity that impacted the shoreline and tidal flat. Methods for geochemical and sedimentological analyses are in Text S1 in the supplemental material.

### Sample collection and sequencing.

Prior to dissection, all live-collected lucinid specimens were temporarily stored and transported in Whirl-Pak bags (Nasco, Fort Atkinson, WI, USA) at ambient temperature ﬁlled with surface water from the habitat (8). Dissection was performed within 30 min or within the same day of collection (explained below), where gill and foot tissues were separated from other body tissues and preserved in RNAlater (8). Dissected gill tissues were divided for molecular and sequence analyses between Clemson University (CU; Clemson, SC, USA) and University of Tennessee—Knoxville (UTK; Knoxville, TN, USA) such that, for each specimen, one fixed gill tissue went to each institution or both gill tissues went to UTK. Valves and the remaining preserved tissues were archived and cataloged at the Museum of Geology at the South Dakota School of Mines and Technology (Rapid City, SD, USA). This study focused on specimens from four of 13 quadrats that were predominantly covered by either seagrass or algae: T20 (20 m), T20 (40 m), T21 (0 m), and T22 (3.5 m) (Fig. 1). *Ctena orbiculata* specimens from these four quadrats used for 16S rRNA gene, metagenomic, and metatranscriptomic analyses at CU were preserved within 30 min of collection. Nucleic acids extraction, fluorometric DNA/RNA quantification, cDNA synthesis, and 16S rRNA gene library preparation from this collection were performed as described in the work of Lim et al. (8). At CU, the V4 region of the 16S rRNA gene from 24 *C. orbiculata* gill tissues, as well as metagenomic libraries prepared from DNA extracted from eight *C. orbiculata* gill samples using NEBNext dsDNA Fragmentase plus the NEBNext Ultra II DNA library prep kit for Illumina or the NEBNext Ultra II FS DNA library prep kit for Illumina (New England Biolabs, Ipswich, MA, USA), were sequenced on the Illumina MiSeq V2 2- by 250-bp platform (San Diego, CA, USA). RNA extracted from 11 *C. orbiculata* gill samples at CU was prepared for metatranscriptomic sequencing, using procedures in the work of Lim et al. (8), on the Illumina HiSeq 4000 2- by 150-bp platform at the Duke Center for Genomic and Computational Biology (Durham, NC, USA). All library concentrations were quantified with the Qubit dsDNA HS assay (Life Technologies, Austin, TX, USA), and their insert sizes were determined with the Agilent 2100 Bioanalyzer (Agilent Technologies, Santa Clara, CA, USA). Specimens of additional *C. orbiculata* individuals (*n* = 3) and other sympatric lucinid species (*n* = 7) from the same four quadrats were also used for only 16S rRNA gene analysis at UTK. These specimens were preserved within the same day of collection. DNA was extracted from the gill tissues of 12 *C. orbiculata* (including 9 of the gill samples fixed by CU), four *L. nassula*, two *A. alba*, and one *Codakia orbicularis* specimen using methods described in the work of Lim et al. (8). 16S rRNA gene V4 region libraries of these samples were prepared and sequenced by Molecular Research LP (Shallowater, TX, USA) using the Illumina MiSeq V3 2- by 300-bp platform.

### Data analyses.

16S rRNA gene sequence reads from CU and UTK were combined, quality trimmed, and processed with MOTHUR v1.41.1 (76) using the pipeline documented in the work of Lim et al. (8). Briefly, all reads were quality trimmed at *Q* = 25 using Mothur’s trim_seq and remove_seq commands and processed according to MOTHUR’s MiSeq SOP available at https://www.mothur.org/wiki/MiSeq_SOP. OTUs clustered at 99% sequence identity were taxonomically classified using the Silva v132 database (28) with 90% bootstrap confidence. The data set was subsampled to 6,676 sequences, which was the number of sequences in the smallest library. OTU relative abundances and Good’s coverage (27) within each sample were calculated using the MOTHUR get.relabund and summary.single commands, as described in the work of Lim et al. (8). Representative sequences of the 10 most abundant OTUs were obtained using MOTHUR’s get.otu rep command, which identifies a representative sequence sharing the smallest maximum or average distance (in the event of a tie) to other sequences classified within the same OTU. Reads from all eight metagenomic libraries were trimmed and assembled individually (8). Additionally, two metagenomic libraries of gill specimens dominated by OTU1 and two libraries of gill specimens dominated by OTU2 were coassembled separately. Read mapping, binning, MAG quality assessment, MAG annotation, and ANI and AAI calculations were performed as detailed in the work of Lim et al. (8). Methods for phylogenetic analyses are outlined in Text S1 in the supplemental material. Bacterial replication rates were estimated using the iRep software (34) by mapping metagenomic reads to representative OTU-specific MAGs (22G, 22B + 4D, 21D, and 4F) with ≥75% completeness and ≤2% contamination, using Bowtie 2 v2.3.4.1 (77) in very sensitive local and dovetail mode and SAMtools v1.7 (78). For metatranscriptomic analyses, a pangenome for each symbiont OTU was created using the method in the work of Lim et al. (8). *De novo* metatranscriptomic assembly, transcript cluster (gene) abundance estimation, count normalization, transcript-to-gene mapping, and transcript annotation were performed using Trinity v2.6.6 (79), Trinotate v3.1.1 (https://trinotate.github.io/), and web and local Blast searches (48), as described in the work of Lim et al. (8). For symbiont abundance estimation, trimmed reads were mapped to representative MAGs using the Bowtie 2 (77) –no-unal –no-mixed –no-discordant –gbar 1000 –end-to-end -k 200 options. Nonchimeric fragments mapped to protein-encoding genes were calculated with featureCounts v1.5.2 (80) using the -c and -p options. DE and functional gene ontology (GO) enrichment analyses were performed on a raw read count matrix processed with Trinity’s remove_batch_effects_from_count_matrix.pl script. Batch-removed read counts of transcript clusters mapped to thioautotrophic symbiont MAGs and those with eukaryotic homologs were analyzed separately using DESeq2 (45), edgeR (46), Reproducibility-Optimized Test Statistic (ROTS) (81), voom (82), and GOSeq (83) software incorporated within Trinity. DESeq2 (45) and edgeR (46) rely on the negative binomial distribution for DE analyses but differ in their normalization and statistical testing methods. ROTS uses properties from gene expression data to optimize a modified *t* statistic that maximizes the overlap of top-ranked genes in group-preserving bootstrapped data sets (81), while voom models the mean-variance relationship of depth-normalized log counts nonparametrically, incorporates it into a precision weight for each individual observation, and then fits a linear model to the weighted data using the *limma* package (84) for DE analyses (82). On the other hand, GOSeq (83) identifies Gene Ontology (GO) (85) terms, extracted from Trinotate’s annotations, that are over- and underrepresented in DE gene data sets predicted by each software program. To identify DE genes and GO terms, a threshold of >2-fold change and <0.05 false-discovery rate (FDR)-adjusted *P* value was applied (86), with the latter corresponding to a false-positive rate of 5% among all statistically significant DE genes. Predictions made by these tools were compared using Venny (87). qPCR methods to verify *mdh* gene expression are described in Text S1.

### Availability of data and materials.

Physical specimen details are available through the iDigBio portal (https://www.idigbio.org/portal/recordsets/db3181c9-48dd-489f-96ab-a5888f5a938c). Sequence data were uploaded to NCBI (48) under the BioProject IDs PRJNA377790 (CU) and PRJNA510358 (UTK). Accession numbers are listed in Table S3.

10.1128/mSystems.00280-19.10 TABLE S3 NCBI accession numbers of raw read and sequence data generated in this study. Download Table S3, DOCX file, 0.01 MB. Copyright © 2019 Lim et al. 2019 Lim et al. This content is distributed under the terms of the Creative Commons Attribution 4.0 International license.
