# Supplementary material for: Extensive Thioautotrophic Gill Endosymbiont Diversity within a Single Ctena orbiculata (Bivalvia: Lucinidae) Population and Implications for Defining Host-Symbiont Specificity and Species Recognition
Source: mSystems. 2019 Aug 27;4(4):e00280-19. doi: 10.1128/mSystems.00280-19 (PMC6712303; doi:10.1128/mSystems.00280-19)
Supplement: TABLE S1 [file mSystems.00280-19-st001.docx]

**Table S1.** Environmental data from Sammy Creek Landing, Sugarloaf Key, Florida. The estimated density of live clams was calculated from the number of live clams recovered from the volume of sediment excavated per quadrat. Dissolved sulfide and dissolved oxygen (DO) measurements are reported in mg/L; dissolved methane concentrations in μg/L; temperature (temp) in Celsius; conductivity (cond) in mS/cm.

| **Quadrat** | **Vol (m^3^)** | **Seagrass or algae species (%)** | **Estimated density of live clam species per m^3^** | | | | | | **Pore water** | | | | |  | **Ocean water** | | | |
| --- | --- | --- | --- | --- | --- | --- | --- | --- | --- | --- | --- | --- | --- | --- | --- | --- | --- | --- |
|  |  |  | ***Ctena orbiculata*** | ***Lucinisca nassula*** | ***Anodontia alba*** | ***Codakia orbicularis*** | ***Parvilucina pectinella*** | ***Radiolucina amianta*** | **Sulfide** | **CH_4_** | **DO** | **Temp** | **pH** |  | **DO** | **Temp** | **pH** | **Cond** |
| T20 (0m) | 0.42 | *Halodule wrightii* (50%)  *Syringodium filiforme* (50%) | 14.1 | 2.5 | 0 | 0 | 0 | 0 | 98.82 | 34.94 | 0.12 | 31.0 | 7.21 |  | 3.36 | 30.2 | 7.90 | 69.2 |
| T20 (10m) | 0.40 | *H. wrightii* (100%) | 2.5 | 0 | 0 | 0 | 0 | 0 | 55.88 | 6.03 | 0.54 | 30.2 | 7.24 |  | 5.40 | 31.2 | 8.15 | 69.7 |
| T20 (20m) | 0.29 | *H. wrightii* (100%) | 20.7 | 3.5 | 0 | 0 | 0 | 0 | 20.35 | 4.29 | 0.51 | 31.1 | 7.27 |  | 5.60 | 31.4 | 8.29 | 69.5 |
| T20 (30m) | 0.30 | *Thalassia testudinum* (60%) Mixed red algae (30%) *Halimeda* spp. algae (10) | 16.7 | 0 | 3.3 | 0 | 0 | 0 | 3.53 | 0.38 | 0.88 | 29.3 | 7.73 |  | 5.49 | 29.3 | 8.02 | 65.3 |
| T20 (40m) | 0.40 | *H. wrightii* (90%)  *T. testudinum* (10%) | 25 | 2.5 | 2.5 | 0 | 0 | 0 | 5.49 | 0.32 | 0.28 | 30.0 | 7.93 |  | 5.56 | 30.5 | 8.22 | 69.0 |
| T20 (50m) | 0.43 | *H. wrightii* (30%)  *T. testudinum* (70%)  trace *Halimeda* spp. algae | 18.6 | 2.3 | 7 | 0 | 0 | 0 | 18.83 | 0.56 | 1.20 | 31.1 | 7.67 |  | 6.59 | 31.9 | 8.14 | 69.6 |
| T21 (0m) | 0.07 | *Halimeda* spp. algae (70%) *Penicillus* spp. (30%)  trace *H. wrightii* | 93.3 | 0 | 0 | 0 | 0 | 0 | 20.00 | 22.41 | 0.69 | 30.5 | 7.44 |  | 2.85 | 30.8 | 7.85 | 67.2 |
| T21 (10m) | 0.10 | *H. wrightii* (100%)  trace *Penicillus* spp. algae | 80 | 10 | 0 | 30 | 0 | 0 | 4.53 | 0.51 | 0.79 | 31.2 | 7.69 |  | 4.63 | 31.3 | 7.96 | 68.5 |
| T21 (20m) | 0.10 | *H. wrightii* (100%) | 20 | 20 | 0 | 130 | 20 | 0 | 0.32 | 0.31 | 1.25 | 31.4 | 7.68 |  | 4.55 | 32.1 | 8.04 | 69.9 |
| T21 (30m) | 0.10 | *H. wrightii* (100%)  trace *Thalassia* spp. | 60 | 30 | 10 | 60 | 0 | 10 | 3.95 | 0.25 | 0.92 | 32.1 | 7.60 |  | 5.66 | 33.4 | 8.17 | 72.3 |
| T21 (40m) | 0.10 | *H. wrightii* (90%)  mixed green algae (10%) | 40 | 0 | 20 | 10 | 0 | 0 | 11.20 | 0.34 | 0.47 | 31.7 | 7.70 |  | 4.11 | 31.6 | 7.93 | 69.0 |
| T21 (50m) | 0.10 | *H. wrightii* (100%)  trace *Halimeda* spp. algae | 40 | 10 | 0 | 50 | 0 | 0 | 2.01 | 0.19 | 0.80 | 32.0 | 7.51 |  | 5.22 | 32.3 | 7.76 | 69.7 |
| T22 (0m) | 0.10 | *Halimeda* spp. algae (100%) trace *H. wrightii* | 140 | 0 | 10 | 10 | 0 | 0 | 8.81 | 0.61 | 1.07 | 33.0 | 7.47 |  | 5.64 | 34.0 | 8.04 | 72.9 |
