# Supplementary material for: Extensive Thioautotrophic Gill Endosymbiont Diversity within a Single Ctena orbiculata (Bivalvia: Lucinidae) Population and Implications for Defining Host-Symbiont Specificity and Species Recognition
Source: mSystems. 2019 Aug 27;4(4):e00280-19. doi: 10.1128/mSystems.00280-19 (PMC6712303; doi:10.1128/mSystems.00280-19)
Supplement: TEXT S1 [file mSystems.00280-19-s0001.docx]

## **Supplementary Information**

## **Supplementary Materials and Methods**

### **Geochemical water and sediment analyses**

Raw and filtered pore waters were collected and analyzed at six locations that corresponded to six quadrat locations within the 10-m^2^ sampling area, and at one nearby inlet open water location. Standard field physicochemistry electrode-based methods were used (1), including pH and conductivity, each with temperature (Fisher Scientific Accumet AP115 and AP75 portable probes, respectively). All analytical geochemistry methods were done as previously described in Green-García and Engel (2). Briefly, pore water was siphoned from approximately 5 – 25 cm deep in the sediments using a stainless steel piezometer, 60 milliliter syringe, and Geotech silicone tubing. Dissolved oxygen was measured from pore water using the Rhodazine D colorimetric method of CHEMetrics (Calverton, VA) with a V-2000 Multi-Analyte Photometer and from open water using a YSI Pro2030 meter. Dissolved sulfide concentrations from the pore waters were also determined using the CHEMetrics methylene blue colorimetric method. Pore and ocean waters were filtered through 0.22-µm Millipore Express™ Sterivex polyethersulfone (PES) membrane filter cartridges into separately prepared HDPE bottles for anions and cations, and alkalinity, and into baked glass vials for total inorganic and dissolved organic carbon (TIC and DOC, respectively) and total nitrogen (TN). The volume of water filtered through each Sterivex filter was recorded and at least two filters were used at each sample location. Cations were preserved with trace metal grade nitric acid in the field. Water samples were maintained at 4^o^C until analysis. Alkalinity was determined within 12 hours by manual titration using 0.1 N sulfuric acid to an endpoint of pH 4.3. Major anions and cations were measured with Dionex (ThermoFisher Scientific) ICS-2000 series ion chromatographs with standards checks accurate within two standard deviations. Concentrations of TIC and DOC were analyzed with a Shimadzu Model TOC-V Total Carbon Analyzer. DOC is the difference between dissolved nonpurgable organic carbon (NPOC) and TIC. The standard used for minimum detection limit was C_8_H_5_KO_4_, and the precision between replicate sample injections was 2% of the relative percent difference (RPD) for DOC >4 mg/L and 5% RPD for DOC <4 mg/L. TN was measured by high temperature catalytic oxidation with chemiluminescence nitrogen detector, with a minimum detection level of 0.01 mg/L. Dissolved gases were measured from raw pore water and open water collected into pre-sterilized, evacuated serum bottles crimp-sealed with a butyl rubber septum. Bottles were filled to atmospheric pressure and maintained at 4^o^C until analysis. Gases were measured using the gas chromatography headspace equilibration method with a SRI Instruments 8610C gas chromatograph with a flame ionization detector, flame photometric detector, and a thermal conductivity detector with a methanizer. Gases of interest included CO_2_, CH_4_, H_2_S, and atmospheric gases O_2_ and N_2_. Sediment samples from within the quadrats were collected in 10 cm increments to a depth of 30 cm and stored frozen until loss-on-ignition (LOI) analysis to estimate water and organic carbon content (in triplicate) and grain size distribution analysis (in duplicate) using GRADISTAT v 8.0 (3).

**Phylogenetic analyses**

Phylogenetic analyses of 16S rRNA gene sequences (K2+G model), Mdh (LG+G+I+F model), and FdhA (LG+G+I model) protein sequences were performed using MEGA7 (4) according to procedures in Lim *et al.* (5). The resulting tree was visualized and annotated with the interactive tree of life (iTOL) v4 online tool (6). Phylogenomic analysis (7) was also conducted using methods described in Lim *et al.* (5), and a concatenated alignment of eight single-copy genes (*dna*G, *nus*A, *pgk*, *rplS*, rpsE, *rpsK*, *rps*M, *smpB*) were used to generate a final maximum likelihood tree with aLRT (approximate likelihood-ratio test) SH-like support values (8) using the protein substitution model LGF for *dnaG*, *nusA*, *pgk*, *rplS*, *rpsM*, RTREVF for *rpsE* and *rpsK*, Dayhoff for *rpsM*, and LG for *smpB*. Host phylogeny was inferred from lucinid marker gene sequences identified in unbinned *C. orbiculata* gill metagenomes using BLAST v2.6.0+’s (9) blastn (10) function and reference sequences retrieved from GenBank (11) via keyword searches. Each identified marker gene set was aligned with BioEdit v7.25’s (12) ClustalW (13) package. The cytochrome *b* gene alignment was analyzed with MEGA7 (4) using the invertebrate mitochondrial genetic code table and the highest scoring Hasegawa-Kishino-Yano model (14) with discrete Gamma distribution modeling of the evolutionary rate differences among sites (5 categories (+G, parameter = 0.8357)). All positions of the gap-free alignment were used for phylogenetic analysis and a maximum likelihood (ML) tree with 1,000 bootstrap replicates was generated. 18S rRNA gene and 28S rRNA gene alignments were concatenated and analyzed with RAxML v7.7.2 (15). Twenty runs of initial tree finding were performed with the GTRGAMMA algorithm and the resulting tree was used for the optimization of each nucleotide model and branch lengths. The GTRCATI model yielded a tree with the highest gamma-based likelihood and was used in the final search for the highest-scoring ML tree from 1,000 bootstrap replicates.

### **qPCR**

Primers targeting the *mdh* genes annotated in OTU1-related MAGs (OTU=operational taxonomic unit; MAG=metagenome-assembled genome; 18F and 694R for cloning; 599F and 694R for qPCR) and OTU2-related MAGs (699F and 1159R for cloning; 699F and 804R for qPCR) were designed using Primer3 (16) in Geneious v8.0 (17) (**Table S2**). qPCR standards were prepared from PCR-cloned *mdh* genes, as detailed in Lim *et al.* (5). cDNA concentrations for qPCR were quantified fluorometrically with the Qubit® ssDNA assay (Life Technologies, Austin, TX, USA). All PCR and qPCR reactions were run on Bio-Rad’s C1000 Touch™ Thermal Cycler (Hercules, CA, USA) under the following conditions: Initial denaturation at 95°C for 3 minutes, 29 (cloning)/34 (qPCR) cycles of denaturation at 95°C for 15 seconds, annealing (**Table S2**) for 30 seconds, extension at 72°C for 30 seconds, followed by elongation at 72°C for 5 minutes. qPCR data was analyzed with the CFX Manager software (Bio-Rad Laboratories) and all copy numbers were normalized to the amount (ng) of input DNA/cDNA.

## **Supplementary Results and Discussion**

### **Other bacterial taxa in the gill microbiome**

Among the ten most abundant OTUs in the 16S rRNA gene dataset, we identified 3±7% average relative abundance of a gammaproteobacterial *Endozoicomonas*-like OTU (OTU7; order Oceanospirillales) most closely related to *E. elysicola* from the gastrointestinal tract of the sea slug *Elysia ornata* (18) (**Figure S1**) in the gill tissues of 19 of 26 *Ctena orbiculata* individuals and all sampled sympatric lucinid individuals (two *Anodontia alba* individuals, four *Lucinisca nassula* individuals, and one *Codakia orbicularis* individual; **Figure 2**). Another *Endocoizomonas*-like OTU (OTU9) was also detected at 1±2% relative abundance in seven *C. orbiculata* individuals, one *A. alba* individual, and three *L. nassula* individuals (**Figure 2**). This OTU was most closely related to gammaproteobacterial phylotypes sequenced from *Loripes orbiculatus* (syn = *Loripes lucinalis*) that were outside the clade of thioautotrophic bivalve symbionts (19) (**Figure S1**). The presence of *Endocoizomonas*-like OTUs in these lucinid gill microbiomes corroborates previous reports of potentially taxonomically and/or functionally-related bacterial members enriched in lucinid gill microbiomes, including *Kistimonas*-like species (order Oceanospirillales) in *Phacoides pectinatus* (5), Gammaproteobacteria *sp*. in *Loripes orbiculatus* (19), and an unclassified rod-shaped taxa in *Euanodontia ovum* (20). *Endozoicomonas* species have also been identified as coral symbionts (21-24), sea squirt commensals (25), and a cobia fish pathogen (26). Two *Spirochaeta*-like OTUs were also among ten most abundant OTUs in lucinid gills. *Spirochaeta*-like OTU8 occurred in the gills of 21 *C. orbiculata* individuals, one *A. alba* individual, two *L. nassula* individuals at 0.8±0.9% average relative abundance (**Figure 2**), and was most closely related to the spirochete symbiont in *Lucinoma aff. kazani* (27) (**Figure S1**). *Spirochaeta*-like OTU10 was present in the gills of two *C. orbiculata* individuals and three *L. nassula* individuals at 2±2% average relative abundance (**Figure 2**), and was most closely related to the *Spirochaeta*-like species in the gills of *P. pectinatus* (5) (**Figure S1**). Besides lucinid bivalves (5, 19, 20, 27), spirochete species have been reported to be symbionts in gutless oligochete worms (28, 29) and an epibiont of a hydrothermal vent worm (30). We did not detect any *Endozoicomonas*-like and *Spirochaeta*-like MAGs in our gill metagenomic assemblies, possibly because of the lack of sequencing coverage on the MiSeq platform. Currently, the roles of Oceanospirillales and spirochete species in the lucinid gill microbiomes remain poorly understood.

### **Urea hydrolysis potential of C. orbiculata symbionts**

*Candidatus* Thiodiazotropha endoloripes (31) and *Ca.* Sedimenticola endophacoides (5) could potentially hydrolyze urea, but only two of eight unbinned *C. orbiculata* gill metagenomes contained two genes homologous to urease subunit gamma from Firmicutes species and urea ABC transporter substrate-binding protein from *Methylomonas* species. Ten transcript clusters encoding allophanate hydrolase, urea carboxylase, urease accessory proteins, and subunits of the urea ABC transporter were detected in two gill metatranscriptomes of OTU1-dominated specimens at average 0.02±0.03 TPM and two metatranscriptomes of OTU2-dominated specimens at average 0.1±0.1 TPM.

### **Phylogenetic analyses of methanol dehydrogenase (mdh) and formate dehydrogenase alpha subunit (fdhA) genes**

OTU4-related Mdh and FdhA protein sequences were the most closely related to those from *Ca.* Thiodiazotropha endolucinida (**Figure S4-S5**). Mdh sequences from lucinid symbionts clustered with sequences from the thioautotrophic gill symbiont of the giant Teredinidae bivalve *Kuphus polythalamia* (32) and a marine purple sulfur bacterium *Thiorhodococcus drewsii* (33). These sequences formed a sister group with sequences from alphaproteobacterial species from the family Rhodospirillaceae, most of which are nitrogen-fixing (**Figure S4**). FdhA sequences from lucinid symbionts were most closely related to the free-living chemolithoautotrophic marine gammaproteobacterial species *Thioalbus denitrificans* (34), the *Kuphus polythalamia* symbiont (32), *Sedimenticola* spp. (35, 36), and methanotrophic gammaproteobacterial species (**Figure S5**). These sequences were, in turn, related to other nitrogen-fixing betaproteobacterial species (**Figure S5**). The distinct phylogenetic distribution of Mdh and FdhA sequences suggests that, although the genes have a common origin in marine habitats, they were likely acquired separately from diazotrophic alphaproteobacterial (Mdh) and betaproteobacterial species (FdhA).

### **Other C1 oxidation genes in C. orbiculata symbionts**

Genes homologous to deltaproteobacterial methanol:corrinoid methyltransferase, methanol methyltransferase corrinoid activation protein, and methyltransferase corrinoid protein were identified in OTU2-related MAGs and transcriptomes (average 1±0.7 TPM), one OTU3-related metatranscriptome (21D from quadrat T21, 0m; 0.008 TPM; **Figure 7c**), and one OTU4-related unbinned metagenomic assembly from specimen 4F from quadrat T20 (40m). These genes potentially convert methanol to a corrinoid protein, which can be subsequently reduced to methane or oxidized to carbon dioxide (37). OTU3-related MAGs and unbinned metagenomic assemblies of all other *C. orbiculata* symbionts included the S-formylglutathione hydrolase (*estD*) gene homologous to that in free-living *Sedimenticola* spp. *estD* converts S-formylglutathione to glutathione and formate in the glutathione-dependent pathway of formaldehyde detoxification (38). This gene was not expressed in the symbiont transcriptomes, but Mollusca-related *estD* was expressed in all gill metatranscriptomes at average 0.9±0.6 TPM. Additionally, OTU3-related MAGs contained oxalyl-CoA decarboxylase (*oxc*) and formyl-CoA transferase (*frc*) genes homologous to protein sequences in Betaproteobacteria and Alphaproteobacteria species. *oxc* converts oxalyl-CoA to formyl-CoA and CO_2_, but *frc* converts formyl-CoA to formate and oxalyl-CoA (39). *oxc* was not expressed in the metatranscriptomes, but a *frc* transcript cluster related to *Escherichia coli* was expressed in the OTU1-dominated gill specimen 22A from the algae-covered quadrat T22 (3.5m) at 0.08 TPM and the OTU2-dominated gill specimen 4D from the seagrass-covered quadrat T20 (40m) at 0.3 TPM. Common C1-related genes identified in *C. orbiculata* and other lucinid symbiont species encoded the bifunctional methylene-H_4_F dehydrogenase/methenyltetrahydrofolate cyclohydrolase (FolD; average 0.2±0.2 TPM in *C. orbiculata* symbionts; not sequenced in *P. pectinatus* gill symbiont (4)), and the respiratory formate dehydrogenase-O (average 1±2 TPM; **Figure 7c**) (37). *folD* likely participates in biosynthesis in these species because the tetrahydrofolate (H_4_F)-dependent formaldehyde oxidation pathway was not completely sequenced in these symbionts (i.e., missing formyl-H_4_F synthetase; **Figure 7b**) (40).

### **Clustered regularly-interspaced short palindromic repeats (CRISPR)-associated genes in C. orbiculata symbionts**

OTU1-related MAGs encoded type I-MYXAN (*Myxococcus xanthus*) CRISPR-associated protein Cas6/Cmx6 that were expressed in two of four OTU1-dominated transcriptomes (average 0.02±0.001TPM) and one OTU2-dominated gill specimen (4D from quadrat T20, 40m; 0.03 TPM). Genes encoding type II CRISPR-associated endonucleases Cas2, Cas6, Cas9 were predicted only in OTU4-related MAGs (not expressed), while one OTU3-related MAG (21D from quadrat T21, 0m) encoded type III-B CRISPR module-associated proteins Cmr1 (0.5 TPM) and Cmr2-6 (not expressed). Inter-taxa genetic differences in CRISPR-Cas system types among *C. orbiculata* symbionts suggest variations in mechanisms of prokaryotic defense against foreign DNA (41). The CRISPR-Cas system is involved in host colonization (42), innate immune avoidance (43), intracellular growth (44), and virulence (45) in other host-microbe interactions, but its role in marine symbiosis has not been investigated and is beyond the scope of this study.

### **Differential expression (DE) analyses across C. orbiculata symbiont communities**

DE analyses were performed to identify candidate genes differentially expressed across C. orbiculata symbiont communities that presumably arise due to differences in taxonomic composition in these communities and/or other factors. Transcript clusters upregulated in OTU1-dominated symbiont communities compared to communities containing significant abundances of other symbiont taxa were involved in bacterial secretion (type VI secretion protein Rhs/TssL/TssA and protein translocase subunit SecD), the transport of sugar, molybdate and an unknown substrate, and other functions. Compared to other taxa, OTU2-related communities preferentially expressed transcript clusters that encoded an efflux transporter of toxic substances (46), a ribosomal small subunit maturation protein GTPase A (47), the signaling molecule diguanylate cyclase facilitating biofilm formation and pathogenesis (48), 5-formyltetrahydrofolate cyclo-ligase regulating purines, thymidylate, and methionine biosynthesis and C1 metabolism (49), heat shock protein, RpoH, and DksA. Commonly predicted Gene Ontology (GO; p<0.05) terms enriched in OTU2-related communities compared to OTU1-dominated communities were associated with nucleotide/nucleoside binding, proteolysis, calcium-transporting ATPase activity, aerobic respiration, and drug response.

Transcript clusters upregulated in OTU3-related communities compared to communities containing significant abundances of other taxa encoded a hypothetical conserved exported protein, flagella-related proteins, acetolactate synthase for branched chain amino acid synthesis (50), calmodulin, cytochrome *c*, a prevent-host-death protein promoting bacteriophage resistance and biofilm formation in *Pseudomonas* species (51), a nitrate ABC transporter, and sulfide dehydrogenase (flavocytochrome c) flavoprotein subunit, among other proteins. GO terms commonly enriched in OTU3-related communities compared to OTU1-dominated communities were related to lysine biosynthesis, flagellar assembly, phosphatase activity, nucleotide binding, membrane components, protein/peptide secretion, and nitrogen compound transport. Although flagellar genes have been implicated in symbiotic host attachment (52) and symbiont-to-host protein export (53, 54), their significance in the lucinid-bacteria symbiosis remains unknown. Similarly, the roles and relevance of differentially expressed amino acid biosynthesis genes in shaping host-symbiont interdependencies on amino acids are unknown.

OTU4-related communities showed upregulation of transcript clusters encoding ribosomal proteins, RNA chaperone Hfq, BAX inhibitor of host apoptosis (55), cytochrome c oxidases involved in aerobic respiration, secretion (type VI secretion, twin-arginine translocation subunit TatA), and stress response (heat shock protein and DnaK). Accordingly, GO terms associated with these functions were enriched in OTU4-related communities compared to OTU1-dominated communities. Upregulated host apoptosis-related symbiotic genes in OTU4-related communities and OTU4-related communities may be involved in the lucinid-bacteria symbiosis. For instance, *Wolbachia*-mediated inhibition of host apoptosis is proposed to effect host wasp transition from facultative parasitism to mutualism (56), but it is unclear whether similar mechanisms exist in the lucinid-bacteria symbiosis.

## **Supplementary References**

1. **Eaton, AD, Clesceri, LS, Rice, EW, Greenberg, AE, Franson, MAH (eds.),** 2005. **Standard methods for the examination of water and wastewater**. American Public Health Association, Washington, D.C.

2. **Green-García, AM, Engel, AS.** 2012. Bacterial diversity of siliciclastic sediments in a *Thalassia testudinum* meadow and the implications for *Lucinisca nassula* chemosymbiosis. Estuar. Coast. Shelf Sci. **112:**153-161. doi: 10.1016/j.ecss.2012.07.010.

3. **Blott, SJ, Pye, K.** 2001. GRADISTAT: a grain size distribution and statistics package for the analysis of unconsolidated sediments. Earth Surf. Process. Landforms. **26:**1237-1248. doi: 10.1002/esp.261.

4. **Kumar, S, Stecher, G, Tamura, K.** 2016. MEGA7: Molecular Evolutionary Genetics Analysis version 7.0 for bigger datasets. Mol. Biol. Evol. **33:**1870-1874. doi: 10.1093/molbev/msw054.

5. **Lim, SJ, Davis, BG, Gill, DE, Walton, J, Nachman, E, Engel, AS, Anderson, LC, Campbell, BJ.** 2019. Taxonomic and functional heterogeneity of the gill microbiome in a symbiotic coastal mangrove lucinid species. Isme J. **13:**902-920. doi: 10.1038/s41396-018-0318-3.

6. **Letunic, I, Bork, P.** 2016. Interactive tree of life (iTOL) v3: an online tool for the display and annotation of phylogenetic and other trees. Nucleic Acids Res. **44:**W242-W245. doi: 10.1093/nar/gkw290.

7. **Seah, B.** 2014. Phylogenomics-tools. GitHub, Inc.

8. **Anisimova, M, Gascuel, O.** 2006. Approximate likelihood-ratio test for branches: a fast, accurate, and powerful alternative. Syst. Biol. **55:**539-552. doi: 10.1080/10635150600755453.

9. **NCBI Resource Coordinators.** 2016. Database resources of the National Center for Biotechnology Information. Nucleic Acids Res. **44:**D7-19. doi: 10.1093/nar/gkv1290.

10. **Altschul, SF, Gish, W, Miller, W, Myers, EW, Lipman, DJ.** 1990. Basic local alignment search tool. J. Mol. Biol. **215:**403-410. doi: 10.1016/S0022-2836(05)80360-2.

11. **Benson, DA, Clark, K, Karsch-Mizrachi, I, Lipman, DJ, Ostell, J, Sayers, EW.** 2014. GenBank. Nucleic Acids Res. D32-D37. doi: 10.1093/nar/gkt1030.

12. **Hall, TA.** 1999. BioEdit: a user-friendly biological sequence alignment editor and analysis program for Windows 95/98/NT. Nucleic Acids Symp. Ser. **41:**95-98.

13. **Thompson, JD, Higgins, DG, Gibson, TJ.** 1994. CLUSTAL W: improving the sensitivity of progressive multiple sequence alignment through sequence weighting, position-specific gap penalties and weight matrix choice. Nucleic Acids Res. **22:**4673-4680.

14. **Hasegawa, M, Kishino, H, Yano, T.** 1985. Dating of the human-ape splitting by a molecular clock of mitochondrial DNA. J. Mol. Evol. **22:**160-174.

15. **Stamatakis, A.** 2006. RAxML-VI-HPC: maximum likelihood-based phylogenetic analyses with thousands of taxa and mixed models. Bioinformatics. **22:**2688-2690. doi: 10.1093/bioinformatics/btl446.

16. **Untergasser, A, Cutcutache, I, Koressaar, T, Ye, J, Faircloth, BC, Remm, M, Rozen, SG.** 2012. Primer3 - new capabilities and interfaces. Nucleic Acids Res. **40:**e115. doi: 10.1093/nar/gks596.

17. **Kearse, M, Moir, R, Wilson, A, Stones-Havas, S, Cheung, M, Sturrock, S, Buxton, S, Cooper, A, Markowitz, S, Duran, C, Thierer, T, Ashton, B, Meintjes, P, Drummond, A.** 2012. Geneious basic: an integrated and extendable desktop software platform for the organization and analysis of sequence data. Bioinformatics. **28:**1647-1649. doi: 10.1093/bioinformatics/bts199.

18. **Kurahashi, M, Yokota, A.** 2007. *Endozoicomonas elysicola* gen. nov., sp. nov., a gamma-proteobacterium isolated from the sea slug *Elysia ornata*. Syst. Appl. Microbiol. **30:**202-206. doi: 10.1016/j.syapm.2006.07.003.

19. **Espinosa, EP, Tanguy, A, Le Panse, S, Lallier, F, Allam, B, Boutet, I.** 2013. Endosymbiotic bacteria in the bivalve *Loripes lacteus*: localization, characterization and aspects of symbiont regulation. J. Exp. Mar. Biol. Ecol. **448:**327-336. doi: 10.1016/j.jembe.2013.07.015.

20. **Ball, AD, Purdy, KJ, Glover, EA, Taylor, JD.** 2009. Ctenidial structure and three bacterial symbiont morphotypes in *Anodontia* (*Euanodontia*) ovum (Reeve, 1850) from the Great Barrier Reef, Australia (Bivalvia: Lucinidae). J. Molluscan Stud. **75:**175-185. doi: 10.1093/mollus/eyp009.

21. **Neave, MJ, Michell, CT, Apprill, A, Voolstra, CR.** 2014. Whole-genome sequences of three symbiotic *Endozoicomonas* strains. Genome Announc. **2:**e00802-14-14. doi: 10.1128/genomeA.00802-14.

22. **Neave, MJ, Rachmawati, R, Xun, L, Michell, CT, Bourne, DG, Apprill, A, Voolstra, CR.** 2017. Differential specificity between closely related corals and abundant *Endozoicomonas* endosymbionts across global scales. Isme J. **11:**186-200. doi: 10.1038/ismej.2016.95.

23. **Neave, MJ, Apprill, A, Ferrier-Pages, C, Voolstra, CR.** 2016. Diversity and function of prevalent symbiotic marine bacteria in the genus *Endozoicomonas*. Appl. Microbiol. Biotechnol. **100:**8315-8324. doi: 10.1007/s00253-016-7777-0.

24. **Ding, JY, Shiu, JH, Chen, WM, Chiang, YR, Tang, SL.** 2016. Genomic insight into the host-endosymbiont relationship of *Endozoicomonas montiporae* CL-33(T) with its coral host. Front. Microbiol. **7:**251. doi: 10.3389/fmicb.2016.00251.

25. **Schreiber, L, Kjeldsen, KU, Funch, P, Jensen, J, Obst, M, Lopez-Legentil, S, Schramm, A.** 2016. *Endozoicomonas* are specific, facultative symbionts of sea squirts. Front. Microbiol. **7:**1042. doi: 10.3389/fmicb.2016.01042.

26. **Mendoza, M, Guiza, L, Martinez, X, Caraballo, X, Rojas, J, Aranguren, LF, Salazar, M.** 2013. A novel agent (*Endozoicomonas elysicola*) responsible for epitheliocystis in cobia *Rachycentrum canadum* larvae. Dis. Aquat. Organ. **106:**31-37. doi: 10.3354/dao02636.

27. **Duperron, S, Fiala-Medioni, A, Caprais, J, Olu, K, Sibuet, M.** 2007. Evidence for chemoautotrophic symbiosis in a Mediterranean cold seep clam (Bivalvia: Lucinidae): comparative sequence analysis of bacterial 16S rRNA, APS reductase and RuBisCO genes. FEMS Microbiol. Ecol. **59:**64-70. doi: 10.1111/j.1574-6941.2006.00194.x.

28. **Blazejak, A, Erseus, C, Amann, R, Dubilier, N.** 2005. Coexistence of bacterial sulfide oxidizers, sulfate reducers, and spirochetes in a gutless worm (Oligochaeta) from the Peru margin. Appl. Environ. Microbiol. **71:**1553-1561. doi: 10.1128/AEM.71.3.1553-1561.2005.

29. **Ruehland, C, Blazejak, A, Lott, C, Loy, A, Erseus, C, Dubilier, N.** 2008. Multiple bacterial symbionts in two species of co-occurring gutless oligochaete worms from Mediterranean sea grass sediments. Environ. Microbiol. **10:**3404-3416. doi: 10.1111/j.1462-2920.2008.01728.x.

30. **Campbell, BJ, Cary, SC.** 2001. Characterization of a novel spirochete associated with the hydrothermal vent polychaete annelid, *Alvinella pompejana*. Appl. Environ. Microbiol. **67:**110-117. doi: 10.1128/AEM.67.1.110-117.2001.

31. **Petersen, JM, Kemper, A, Gruber-Vodicka, H, Cardini, U, van, dG, Kleiner, M, Bulgheresi, S, MuBmann, M, Herbold, C, Seah, BKB, Antony, CP, Liu, D, Belitz, A, Weber, M.** 2016. Chemosynthetic symbionts of marine invertebrate animals are capable of nitrogen fixation. Nat. Microbiol. **2:**16195. doi: 10.1038/nmicrobiol.2016.195.

32. **Distel, DL, Altamia, MA, Lin, Z, Shipway, JR, Han, A, Forteza, I, Antemano, R, Limbaco, MGJP, Tebo, AG, Dechavez, R, Albano, J, Rosenberg, G, Concepcion, GP, Schmidt, EW, Haygood, MG.** 2017. Discovery of chemoautotrophic symbiosis in the giant shipworm *Kuphus polythalamia* (Bivalvia: Teredinidae) extends wooden-steps theory. Proc. Natl. Acad. Sci. U. S. A. **114:**E3652-E3658. doi: 10.1073/pnas.1620470114.

33. **Zaar, A, Fuchs, G, Golecki, JR, Overmann, J.** 2003. A new purple sulfur bacterium isolated from a littoral microbial mat, *Thiorhodococcus drewsii* sp. nov. Arch. Microbiol. **179:**174-183. doi: 10.1007/s00203-002-0514-3.

34. **Park, S, Pham, VH, Jung, M, Kim, S, Kim, J, Roh, D, Rhee, S.** 2011. *Thioalbus denitrificans* gen. nov., sp. nov., a chemolithoautotrophic sulfur-oxidizing gammaproteobacterium, isolated from marine sediment. Int. J. Syst. Evol. Microbiol. **61:**2045-2051. doi: 10.1099/ijs.0.024844-0.

35. **Carlstrom, CI, Loutey, DE, Wang, O, Engelbrektson, A, Clark, I, Lucas, LN, Somasekhar, PY, Coates, JD.** 2015. Phenotypic and genotypic description of *Sedimenticola selenatireducens* strain CUZ, a marine (per)chlorate-respiring gammaproteobacterium, and its close relative the chlorate-respiring *Sedimenticola* strain NSS. Appl. Environ. Microbiol. **81:**2717-2726. doi: 10.1128/AEM.03606-14.

36. **Flood, BE, Jones, DS, Bailey, JV.** 2015. Complete genome sequence of *Sedimenticola thiotaurini* strain SIP-G1, a polyphosphate- and polyhydroxyalkanoate-accumulating sulfur-oxidizing gammaproteobacterium isolated from salt marsh sediments. Genome Announc. **3:**e00671-15. doi: 10.1128/genomeA.00671-15.

37. **Abaibou, H, Pommier, J, Benoit, S, Giordano, G, Mandrand-Berthelot, M.** 1995. Expression and characterization of the *Escherichia coli* *fdo* locus and a possible physiological role for aerobic formate dehydrogenase. J. Bacteriol. **177:**7141-7149.

38. **Chen, NH, Djoko, KY, Veyrier, FJ, McEwan, AG.** 2016. Formaldehyde stress responses in bacterial pathogens. Front. Microbiol. **7:**257. doi: 10.3389/fmicb.2016.00257.

39. **Azcarate-Peril, M, Bruno-Barcena, JM, Hassan, HM, Klaenhammer, TR.** 2006. Transcriptional and functional analysis of oxalyl-coenzyme A (CoA) decarboxylase and formyl-CoA transferase genes from *Lactobacillus acidophilus*. Appl. Environ. Microbiol. **72:**1891-1899. doi: 10.1128/AEM.72.3.1891-1899.2006.

40. **Vorholt, JA.** 2002. Cofactor-dependent pathways of formaldehyde oxidation in methylotrophic bacteria. Arch. Microbiol. **178:**239-249. doi: 10.1007/s00203-002-0450-2.

41. **Makarova, KS, Haft, DH, Barrangou, R, Brouns, SJ, Charpentier, E, Horvath, P, Moineau, S, Mojica, FJ, Wolf, YI, Yakunin, AF, van der Oost, J, Koonin, EV.** 2011. Evolution and classification of the CRISPR-Cas systems. Nat. Rev. Microbiol. **9:**467-477. doi: 10.1038/nrmicro2577.

42. **Veesenmeyer, JL, Andersen, AW, Lu, X, Hussa, EA, Murfin, KE, Chaston, JM, Dillman, AR, Wassarman, KM, Sternberg, PW, Goodrich-Blair, H.** 2014. NilD CRISPR RNA contributes to *Xenorhabdus nematophila* colonization of symbiotic host nematodes. Mol. Microbiol. **93:**1026-1042. doi: 10.1111/mmi.12715.

43. **Sampson, TR, Saroj, SD, Llewellyn, AC, Tzeng, YL, Weiss, DS.** 2013. A CRISPR/Cas system mediates bacterial innate immune evasion and virulence. Nature. **497:**254-257. doi: 10.1038/nature12048.

44. **Gunderson, FF, Cianciotto, NP.** 2013. The CRISPR-associated gene *cas2* of *Legionella pneumophila* is required for intracellular infection of amoebae. MBio. **4:**e00074-13. doi: 10.1128/mBio.00074-13.

45. **Louwen, R, Staals, RH, Endtz, HP, van Baarlen, P, van der Oost, J.** 2014. The role of CRISPR-Cas systems in virulence of pathogenic bacteria. Microbiol. Mol. Biol. Rev. **78:**74-88. doi: 10.1128/MMBR.00039-13.

46. **Anes, J, McCusker, MP, Fanning, S, Martins, M.** 2015. The ins and outs of RND efflux pumps in *Escherichia coli*. Front. Microbiol. **6:**587. doi: 10.3389/fmicb.2015.00587.

47. **Goto, S, Kato, S, Kimura, T, Muto, A, Himeno, H.** 2011. RsgA releases RbfA from 30S ribosome during a late stage of ribosome biosynthesis. Embo J. **30:**104-114. doi: 10.1038/emboj.2010.291.

48. **Schirmer, T.** 2016. C-di-GMP synthesis: structural aspects of evolution, catalysis and regulation. J. Mol. Biol. **428:**3683-3701. doi: 10.1016/j.jmb.2016.07.023.

49. **Meier, C, Carter, LG, Winter, G, Owens, RJ, Stuart, DI, Esnouf, RM.** 2007. Structure of 5-formyltetrahydrofolate cyclo-ligase from *Bacillus anthracis* (BA4489). Acta Crystallogr. Sect. F. Struct. Biol. Cryst. Commun. **63:**168-172. doi: 10.1107/S1744309107007221.

50. **Chipman, D, Barak, Z, Schloss, JV.** 1998. Biosynthesis of 2-aceto-2-hydroxy acids: acetolactate synthases and acetohydroxyacid synthases. Biochimica Et Biophysica Acta (BBA) - Protein Structure and Molecular Enzymology. **1385:**401-419. doi: 10.1016/S0167-4838(98)00083-1.

51. **Petrova, OE, Schurr, JR, Schurr, MJ, Sauer, K.** 2011. The novel *Pseudomonas aeruginosa* two-component regulator BfmR controls bacteriophage-mediated lysis and DNA release during biofilm development through PhdA. Mol. Microbiol. **81:**767-783. doi: 10.1111/j.1365-2958.2011.07733.x.

52. **Millikan, DS, Ruby, EG.** 2004. *Vibrio fischeri* flagellin A is essential for normal motility and for symbiotic competence during initial squid light organ colonization. J. Bacteriol. **186:**4315-4325. doi: 10.1128/JB.186.13.4315-4325.2004.

53. **Maezawa, K, Shigenobu, S, Taniguchi, H, Kubo, T, Aizawa, S, Morioka, M.** 2006. Hundreds of flagellar basal bodies cover the cell surface of the endosymbiotic bacterium *Buchnera aphidicola* sp. strain APS. J. Bacteriol. **188:**6539-6543. doi: 10.1128/JB.00561-06.

54. **Toft, C, Fares, MA.** 2008. The evolution of the flagellar assembly pathway in endosymbiotic bacterial genomes. Mol. Biol. Evol. **25:**2069-2076. doi: 10.1093/molbev/msn153.

55. **Hemrajani, C, Berger, CN, Robinson, KS, Marches, O, Mousnier, A, Frankel, G.** 2010. NleH effectors interact with Bax inhibitor-1 to block apoptosis during enteropathogenic *Escherichia coli i*nfection. Proc. Natl. Acad. Sci. U. S. A. **107:**3129-3134. doi: 10.1073/pnas.0911609106.

56. **Pannebakker, BA, Loppin, B, Elemans, CP, Humblot, L, Vavre, F.** 2007. Parasitic inhibition of cell death facilitates symbiosis. Proc. Natl. Acad. Sci. U. S. A. **104:**213-215. doi: 10.1073/pnas.0607845104.

## 
