# Supplementary figures and images for: Extensive Thioautotrophic Gill Endosymbiont Diversity within a Single Ctena orbiculata (Bivalvia: Lucinidae) Population and Implications for Defining Host-Symbiont Specificity and Species Recognition
Source: mSystems. 2019 Aug 27;4(4):e00280-19. doi: 10.1128/mSystems.00280-19 (PMC6712303; doi:10.1128/mSystems.00280-19)

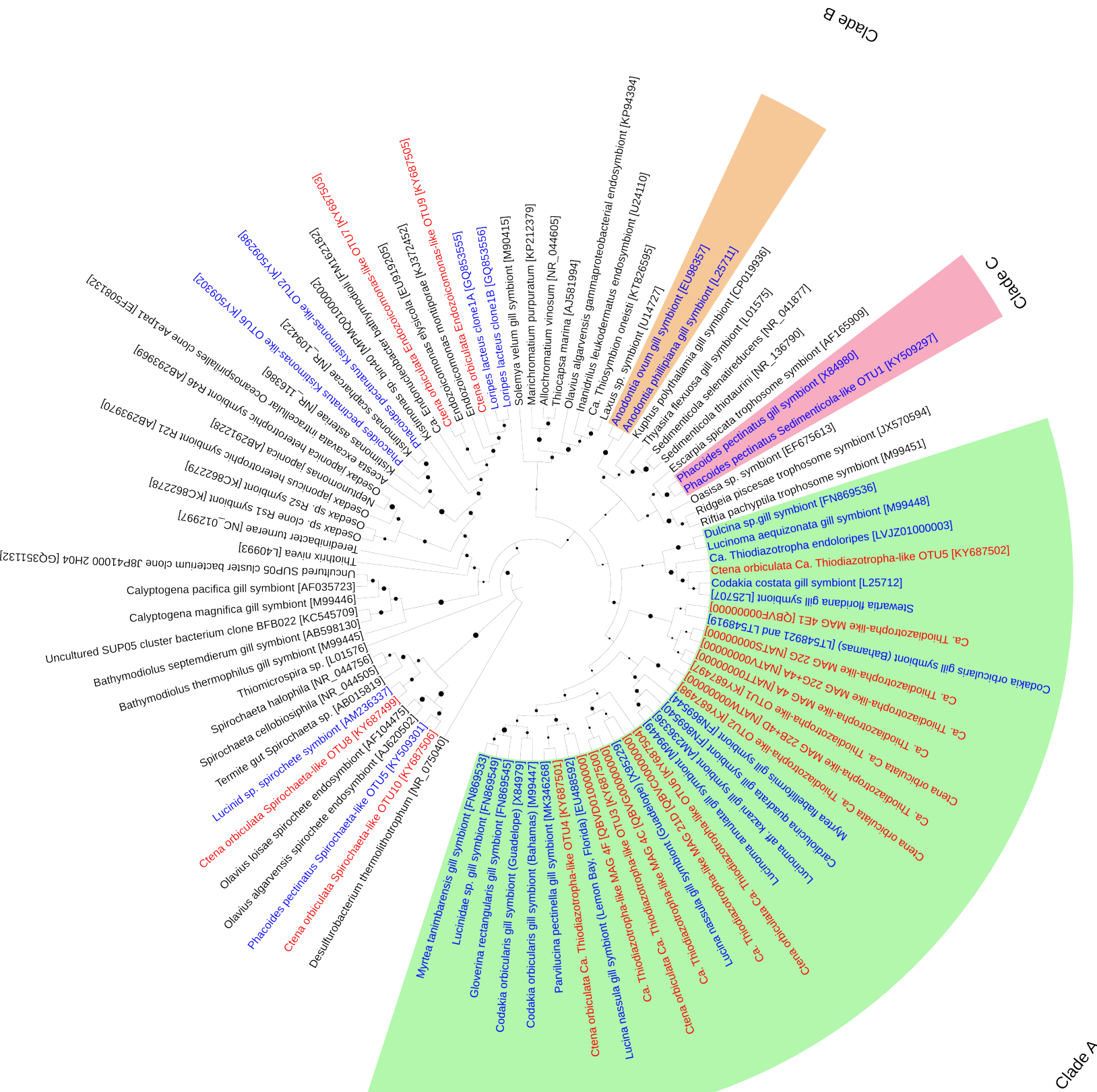

Supplement: FIG S1 [file mSystems.00280-19-sf001.pdf]

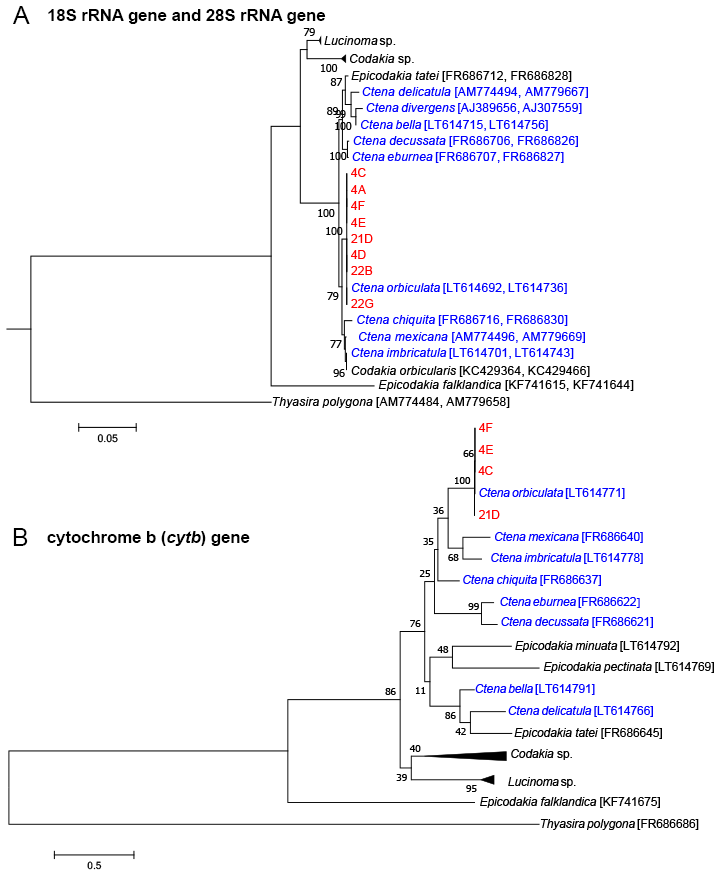

Supplement: FIG S2 [file mSystems.00280-19-sf002.tif]

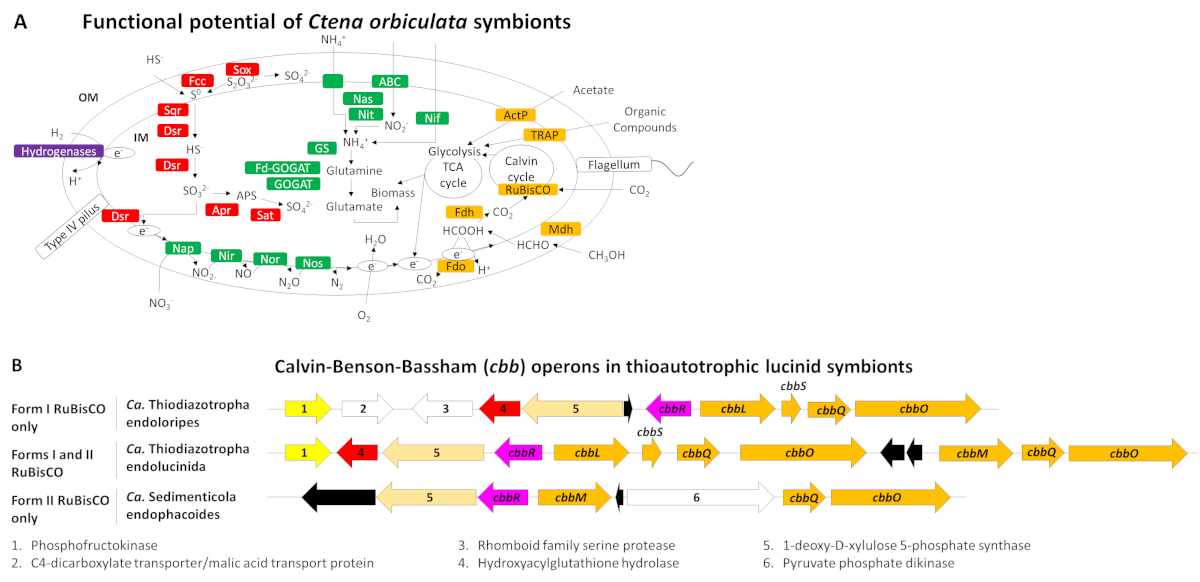

Supplement: FIG S3 [file mSystems.00280-19-sf003.tif]

PQQ-dependent methanol dehydrogenase

Unrooted bootstrap consensus tree

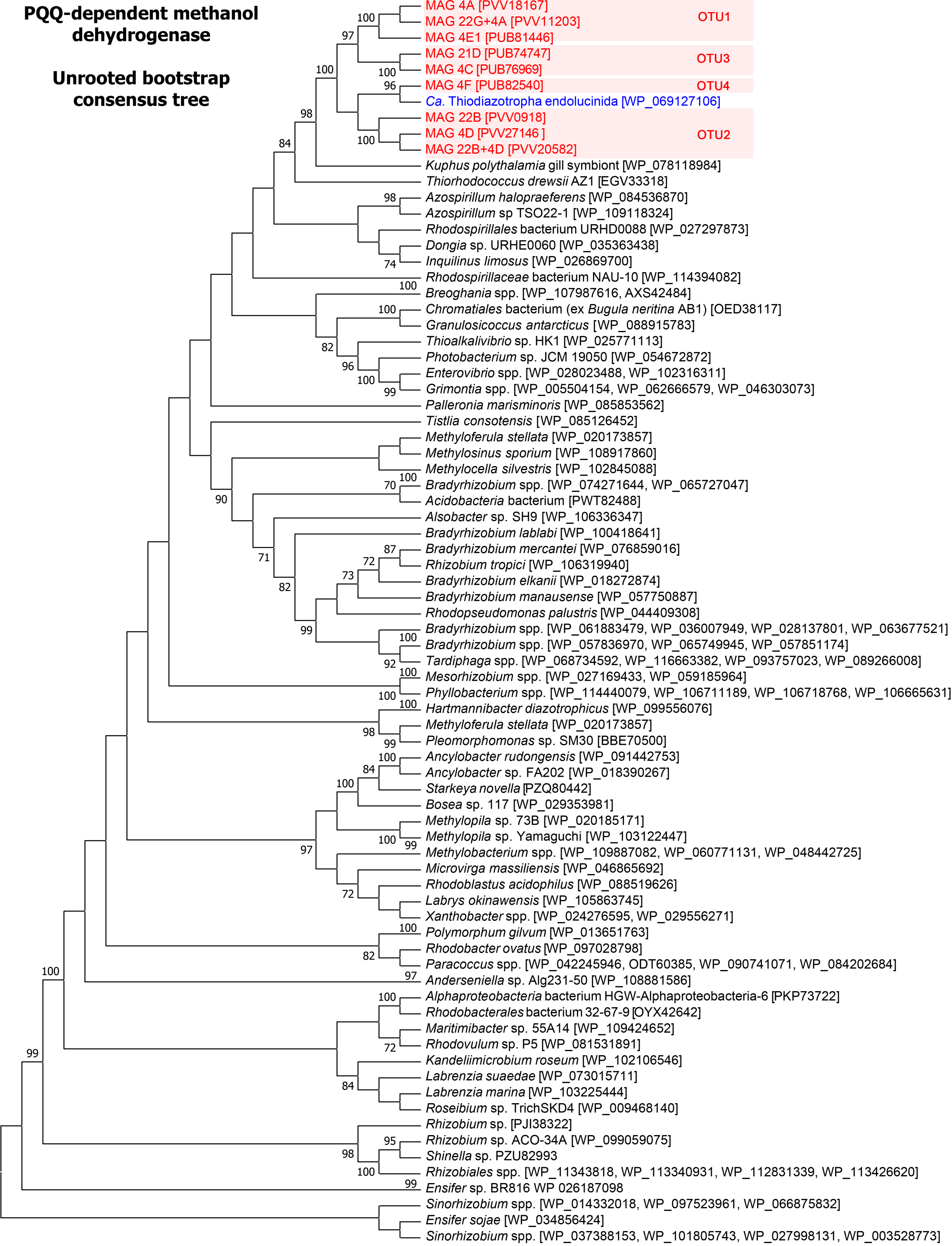

Supplement: FIG S4 [file mSystems.00280-19-sf004.pdf]

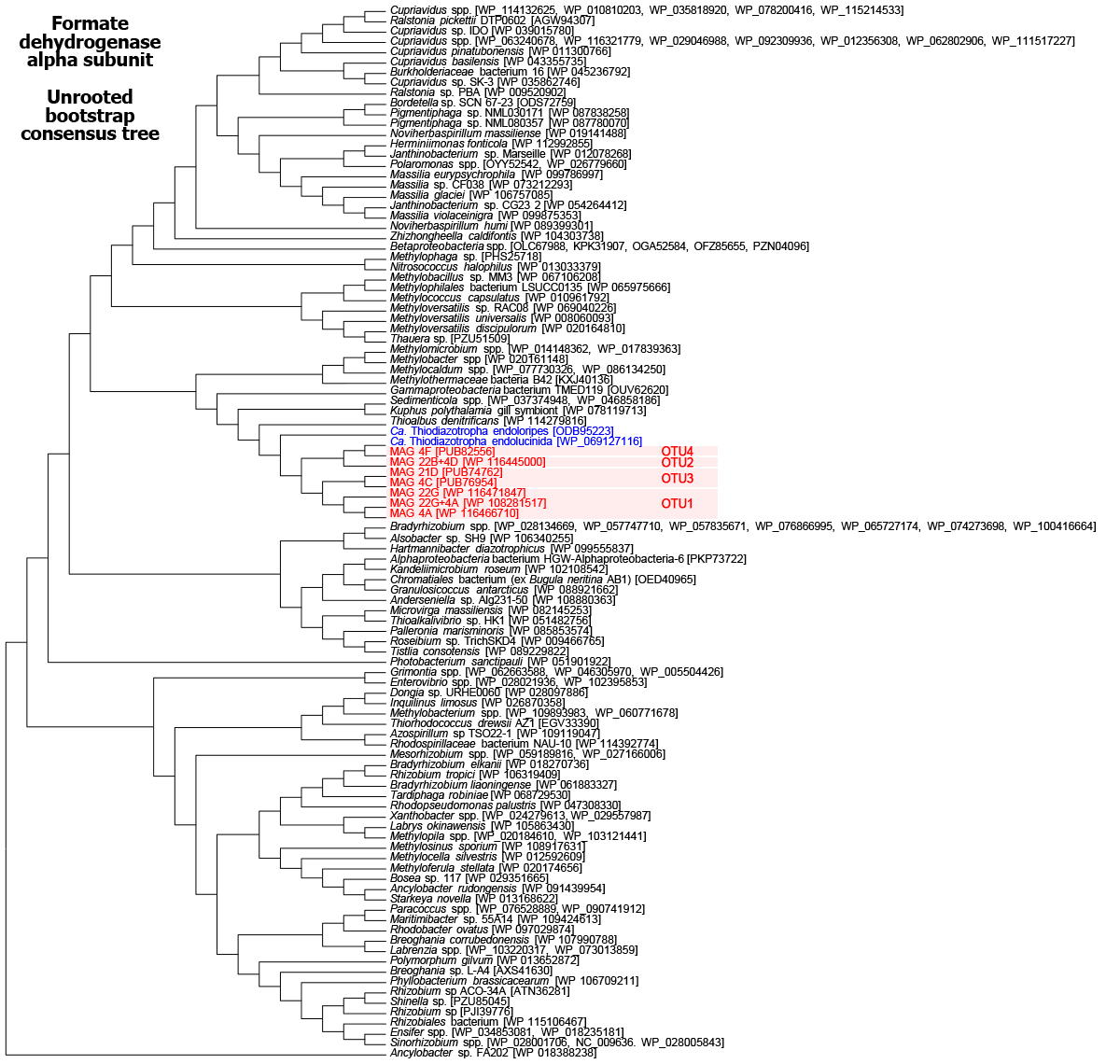

Supplement: FIG S5 [file mSystems.00280-19-sf005.tif]

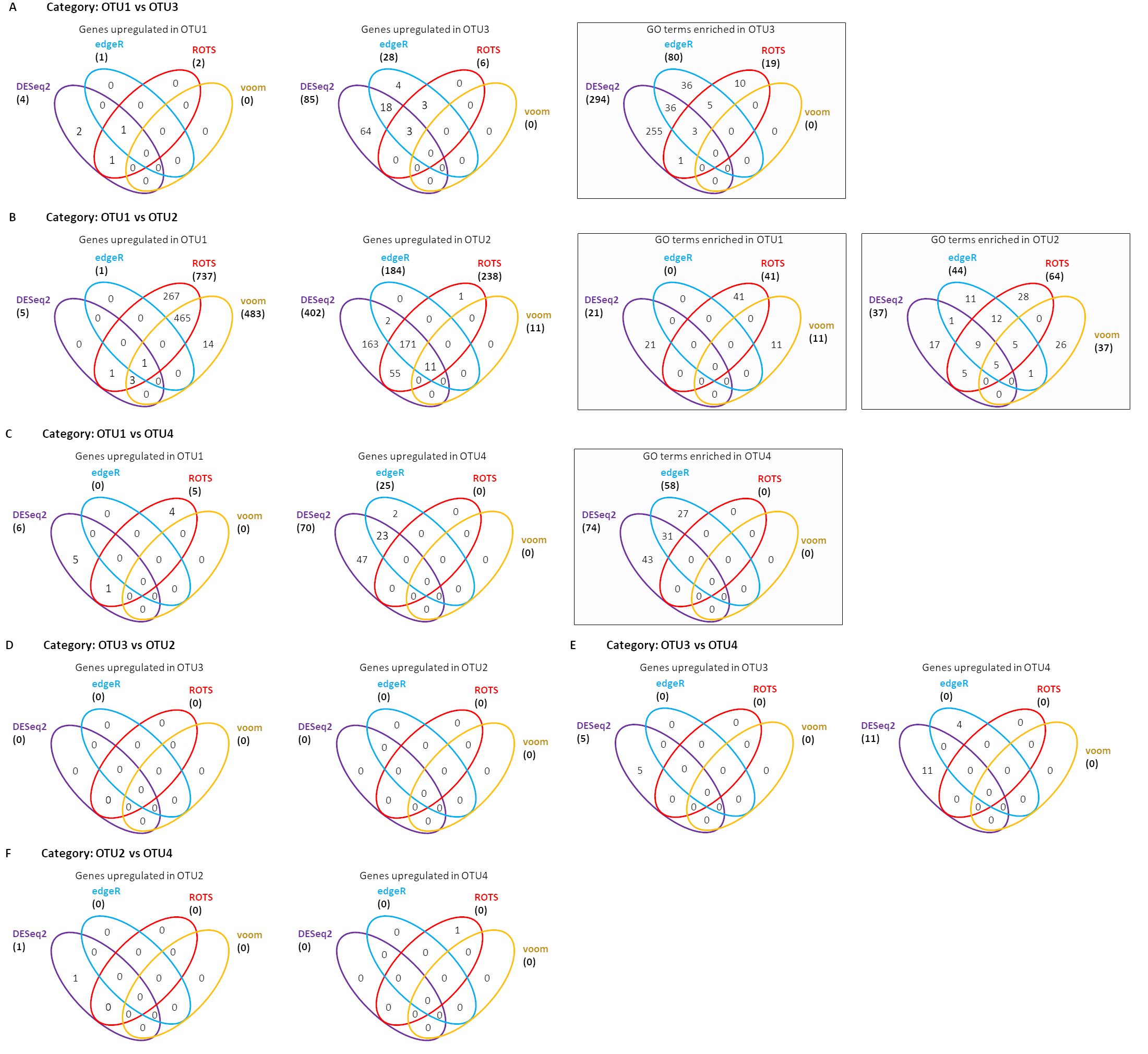

Supplement: FIG S6 [file mSystems.00280-19-sf006.tif]
