# Supplementary material for: Extensive Thioautotrophic Gill Endosymbiont Diversity within a Single Ctena orbiculata (Bivalvia: Lucinidae) Population and Implications for Defining Host-Symbiont Specificity and Species Recognition
Source: mSystems. 2019 Aug 27;4(4):e00280-19. doi: 10.1128/mSystems.00280-19 (PMC6712303; doi:10.1128/mSystems.00280-19)
Supplement: TABLE S2 [file mSystems.00280-19-st002.docx]

**Table S2.** List of PCR and qPCR primers used in this study.

| **Primer** | **Annealing temperature** | **Sequence (5’->3’)** |
| --- | --- | --- |
| *mdh* OTU1 18F | 57.8°C | TACCCTGCTCGATCCCAAGA |
| *mdh* OTU1 599F | 59.5°C | CATCCTACTCGCCACGTACC |
| *mdh* OTU1 694R | 59.5°C (PCR for cloning)/57.8°C (qPCR) | GTTGACCCGCGGTATAGGAG |
| *mdh* OTU2 699F | 59.5°C (PCR for cloning)/55.2°C (qPCR) | GAAGACCACCCATCTTGGCA |
| *mdh* OTU2 804R | 59.5°C | GGAGTACCAACCCCAAGTGG |
| *mdh* OTU2 1159R | 55.2°C | GGCCTGTCTTCATGTCCACA |
