# Supplementary material for: Extensive Thioautotrophic Gill Endosymbiont Diversity within a Single Ctena orbiculata (Bivalvia: Lucinidae) Population and Implications for Defining Host-Symbiont Specificity and Species Recognition
Source: mSystems. 2019 Aug 27;4(4):e00280-19. doi: 10.1128/mSystems.00280-19 (PMC6712303; doi:10.1128/mSystems.00280-19)
Supplement: TABLE S3 [file mSystems.00280-19-st003.docx]

**Table S3.** NCBI accession numbers of raw read and sequence data generated in this study.

| **Database** | **Bioproject ID** | **Accession numbers** | **Dataset description** |
| --- | --- | --- | --- |
| Sequence Read Archive (SRA) | PRJNA377790 | SRR5873713-SRR5873738; SRR7235714; SRR7235722- SRR7235725; SRR7235728- SRR7235730 | Amplicon-sequenced read data (V4 region of 16S rRNA gene) from Clemson University |
|  | PRJNA510358 | SRX5170306-SRX5170454 | Amplicon-sequenced read data (V4 region of 16S rRNA gene) from University of Tennessee-Knoxville |
|  |  |  |  |
|  |  | SRR5872870-SRR5872873; SRS3349532-SRS3349535 | Metagenomic read data |
|  |  | SRR7235715-SRR7235721; SRR7235726-SRR7235727; SRR7235731 | Metatranscriptomic read data |
| GenBank | PRJNA377790 | KY687497-KY687506 | Sequences of top ten most abundant OTUs |
|  | PRJNA377790 | NATR00000000-NATW00000000; QBVC00000000-QBVG00000000 | Metagenome-assembled genomes (MAGs) |
